# Epidemiology of diagnostic errors in pediatric emergency departments using electronic triggers

**DOI:** 10.1111/acem.15087

**Published:** 2025-01-15

**Authors:** Prashant Mahajan, Emily White, Kathy Shaw, Sarah J. Parker, James Chamberlain, Richard M. Ruddy, Elizabeth R. Alpern, Jacqueline Corboy, Andrew Krack, Brandon Ku, Daphne Morrison Ponce, Asha S. Payne, Elizabeth Freiheit, Gregor Horvath, Giselle Kolenic, Michele Carney, Nicole Klekowski, Karen J. O'Connell, Hardeep Singh

**Affiliations:** ^1^ University of Michigan Ann Arbor Michigan USA; ^2^ Children's Hospital of Philadelphia Philadelphia Pennsylvania USA; ^3^ Children's National Health System Washington DC USA; ^4^ University of Cincinnati College of Medicine Cincinnati Ohio USA; ^5^ Ann and Robert H. Lurie Children's Hospital of Chicago Chicago Illinois USA; ^6^ Naval Medical Center Portsmouth Portsmouth Virginia USA; ^7^ Center for Innovations in Quality, Effectiveness and Safety Michael E. DeBakey Veterans Affairs Medical Center and Baylor College of Medicine Houston Texas USA

**Keywords:** diagnostic error, electronic trigger, emergency department, patient safety, pediatrics

## Abstract

**Objectives:**

We applied three electronic triggers to study frequency and contributory factors of missed opportunities for improving diagnosis (MOIDs) in pediatric emergency departments (EDs): return visits within 10 days resulting in admission (Trigger 1), care escalation within 24 h of ED presentation (Trigger 2), and death within 24 h of ED visit (Trigger 3).

**Methods:**

We created an electronic query and reporting template for the triggers and applied them to electronic health record systems of five pediatric EDs for visits from 2019. Clinician reviewers manually screened identified charts and initially categorized them as “unlikely for MOIDs” or “unable to rule out MOIDs” without a detailed chart review. For the latter category, reviewers performed a detailed chart review using the Revised Safer Dx Instrument to determine the presence of a MOID.

**Results:**

A total of 2937 ED records met trigger criteria (Trigger 1 1996 [68%], Trigger 2 829 [28%], Trigger 3 112 [4%]), of which 2786 (95%) were categorized as unlikely for MOIDs. The Revised Safer Dx Instrument was applied to 151 (5%) records and 76 (50%) had MOIDs. The overall frequency of MOIDs was 2.6% for the entire cohort, 3.0% for Trigger 1, 1.9% for Trigger 2, and 0% for Trigger 3. Brain lesions, infections, or hemorrhage; pneumonias and lung abscess; and appendicitis were the top three missed diagnoses. The majority (54%) of MOIDs cases resulted in patient harm. Contributory factors were related to patient‐provider (52.6%), followed by patient factors (21.1%), system factors (13.2%), and provider factors (10.5%).

**Conclusions:**

Using electronic triggers with selective record review is an effective process to screen for harmful diagnostic errors in EDs: detailed review of 5% of charts revealed MOIDs in half, of which half were harmful to the patient. With further refining, triggers can be used as effective patient safety tools to monitor diagnostic quality.

## INTRODUCTION

The National Academies of Science, Engineering, and Medicine (NASEM)'s report, “Improving Diagnosis in Health Care,” highlighted diagnostic safety as an area of opportunity in the patient safety movement.^1^ Diagnostic decision making in busy and time‐pressured emergency departments (EDs) is particularly challenging and poorly understood.^1^ This challenge is amplified in emergency care for children, who often receive care in settings that may not be adequately equipped to meet their specific needs.^2^ About 5% of ambulatory visits among adults involve diagnostic errors, which, if extrapolated to approximately 30 million pediatric ED visits, could result in 1.5 million instances of diagnostic errors annually.^3^ However, there is limited understanding of the epidemiology of diagnostic errors in pediatric ED encounters.

Triggers, which are defined as a specific set of clues used to flag health records of patients at higher risk of harm, have been used to investigate diagnostic errors in EDs.^4^ Using robust consensus methods, we previously developed a pediatric ED‐relevant diagnostic error model and identified three electronic health record (EHR)‐based triggers to identify children with potential diagnostic errors.^5^, ^6^ These electronic triggers included return ED visits with inpatient hospitalization within 10 days of the index ED visit, transfer of an admitted patient to a higher level of care within 24 h of hospitalization, and death within 24 h of ED visit. The Revised Safer Dx Instrument can assess records identified using triggers to determine if a diagnostic error, defined as missed opportunities for improving diagnosis (MOID), has occurred; identify causative factors; and determine the level of patient harm.^7^, ^8^, ^9^, ^10^, ^11^

In this study we determined the frequency, clinical patterns, contributing factors, and associated patient harm from a cross‐section of MOIDs detected using three EHR‐based triggers and the Revised Safer Dx Instrument in five pediatric EDs during a 12‐month period.

## METHODS

### Study setting

We conducted a retrospective, cross‐sectional study involving patients ≤21 years of age presenting to one of five participating pediatric ED systems in 2019. Participating sites are free‐standing pediatric EDs each part of an academic medical center in the Midwest or Eastern United States. The sites have robust EHR systems and were chosen because they have substantial expertise in pediatric ED safety and quality. Together the five sites had approximately 388,000 annual visits with an average admission rate of 18.4% (an average of 16.8% were to a general inpatient unit and 1.6% were to an intensive care unit [ICU]; see Data S1 for individual site details). The study was reviewed as expedited or exempt by the institutional review board at each participating site.

### Trigger criteria

As part of this project, we developed a comprehensive list of triggers that have been used to study safety and quality in the ED.^6^ We arrived at a consensus using a multidisciplinary panel of experts on which triggers could be used to investigate the epidemiology of diagnostic errors. We tested the previously identified EHR‐based triggers to screen a retrospective cohort of patient presentations at the participating pediatric EDs.^6^ Patient records were eligible for review if the patient presenting was ≤21 years of age and met at least one of the following trigger criteria: unscheduled return visit with admission within 10 days (Trigger 1); care escalation to ICU within 24 h after inpatient admission (Trigger 2); of death within 24 h of ED arrival or admission (Trigger 3); see Data S2 for the complete data query used to identify cases for chart review and to assess trigger performance). Elements of interest for the electronic triggers, as applicable, include the following: date/time of presentation, age, sex, reason for visit, clinical impression (ICD‐10), date/time of ED disposition, admitting unit, admitting and discharge diagnoses (ICD‐10), length of stay, hospital disposition, date/time of final hospital discharge, and date/time of death.

### Test set and data collection

We applied the electronic trigger algorithms to EHR data from August 2018 at each site to test the accuracy of the triggers in identifying cases with potential MOIDs. The reviewers received detailed training (including a formal in‐person training program covering case scenarios, in‐depth explanations regarding study procedures, data entry procedures, and quality assurance) and both reviewers at each site tested data collection on the same 1 month of charts to ensure consistency in the process. We collected feedback from reviewers to refine trigger query criteria and improve our chances of finding MOID cases.

We applied the refined triggers to the EHR systems to screen all pediatric ED visits at the participating sites from January 1 to December 31, 2019. We used EHR data of patient presentations to identify potential MOIDs in the pediatric ED, contributing factors and potential harm based on the review of records by two trained pediatric emergency physicians at each participating site.^9^, ^12^ To determine the frequency of MOIDs among the trigger populations, we assessed the clinical care provided to eligible patients during their ED visit and any trigger‐specific subsequent hospitalization. Reviewers used standardized electronic case report forms to record their findings.

Charts identified by one of the triggers were initially manually screened by one of the two site reviewers to determine whether they should be excluded from further review based on the assessment that they were very unlikely to indicate a MOID. Reasons for exclusion from review for MOIDs included progression of the illness not likely to be an error (e.g., upper respiratory infection that progressed to bronchiolitis), unrelated return visits, medically appropriate care escalation over course of illness, mental health complaints, dead on arrival or active cardiopulmonary resuscitation on arrival, and incorrect or limitation of the trigger programming. Charts not excluded by this process were reviewed for the possibility of a MOID using the Revised Safer Dx Instrument, which captures chart review findings on a 13‐item survey and considers such aspects as history, physical findings, and listing of differential diagnoses. Each item is rated on a scale of 1–7, with scores of 5–7 on the summative item 13 indicating the presence of a probable diagnostic missed opportunity.^11^ A MOID existed if adequate data from the chart review suggested the final correct diagnosis was already present at the index ED visit or if documented abnormal findings at the index visit should have prompted additional evaluation that would have revealed the correct, ultimate diagnosis. If the chart received a score less than 4, the chart was not considered a MOID. If the chart under review received a score of 4, the second reviewer examined the chart, and the two reviewers came to a consensus on a final score. If the two reviewers could not come to an agreement or if the agreed‐upon score remained at 4, the investigative team provided final adjudication of whether the chart represented a MOID. All charts considered to be MOIDs underwent further review to determine factors that contributed to the missed opportunity in the diagnostic process (see Data S3 for a data flow chart).

### Sample size justification

The three EHR‐based triggers at the five participating EDs identified 7615 Trigger 1 records, 830 Trigger 2 records, and 113 Trigger 3 records. Based on prior literature,^13^ we assumed a prevalence of MOIDs of 6% in the Trigger 1 group, 7.5% in the Trigger 2 group, and 0% in the Trigger 3 group. We determined it was necessary to review 507 Trigger 1 charts, 800 Trigger 2 charts, and 100 Trigger 3 charts to detect a prevalence of 6% (±2%) with 80% power and an alpha of 0.05 for each trigger. Because of the large number of Trigger 1 records, we randomly selected 400 of these charts per site for review using the Excel RAND function. All Trigger 2 and Trigger 3 records were reviewed.

### Data classification schema

#### Diagnosis classification

To provide meaningful groups for analysis, patient diagnoses were categorized using the Clinical Classifications Software Refined (CCSR) for ICD‐10‐CM diagnoses (a software tool developed as part of the Healthcare Cost and Utilization Project [HCUP], a federal–state–industry partnership sponsored by the Agency for Healthcare Research and Quality [AHRQ]).^14^

#### Reason for visit

The patient's reason for visit was coded according to the 2020 National Hospital Ambulatory Medical Care Survey (NHAMCS) public‐use documentation reason categories.^15^

#### Contributing factors

Reviewers assessed contributing factors to MOIDs using predefined category selections on a standardized case report form that was adapted from prior research in primary care.^16^ The full list of contributing factors used is provided in Data S4.

#### Harm

Potential severity of injury associated with the delay or missed diagnosis was assessed according to the National Coordinating Council for Medication Error Reporting and Prevention (NCC MERP).^17^

### Statistical analysis

All reviewed charts were used to calculate the proportion of MOIDs. All MOIDs identified in the process of chart review were used to generate descriptive statistics regarding process dysfunctions and patient harm. Based on our assumptions, we expected to identify 182 MOIDs ((400 × 5 × 0.06) + (830 × 0.075) + (113 × 0)). All reviewed charts were included in the evaluation of simple and multivariable associations of patient and clinical characteristics with the presence of MOIDs among charts identified by one of the triggers. The measures of interest were summarized with appropriate descriptive statistics and included patient age measured continuously and categorically, sex, race, reason for visit, diagnostic category, insurance provider, length of ED visit, and visit timing. Measures were summarized for the overall sample and stratified by trigger and presence of MOID. Distributional differences in measures of interest between the stratification factors were assessed with independent samples *t*‐tests and chi‐square tests of independence. Simple and multivariable logistic regressions assessed associations among study variables including age, sex, race, insurance, arrival (time, day, month), visit length of stay, and presenting symptoms and the probability of having a MOID based on clinical relevance. We used a two‐sided significance level of 5% for all tests; no adjustments were made to control for Type 1 error rates. All statistical analyses were programmed in R (R Core Team, 2021).

## RESULTS

**FIGURE 1:**
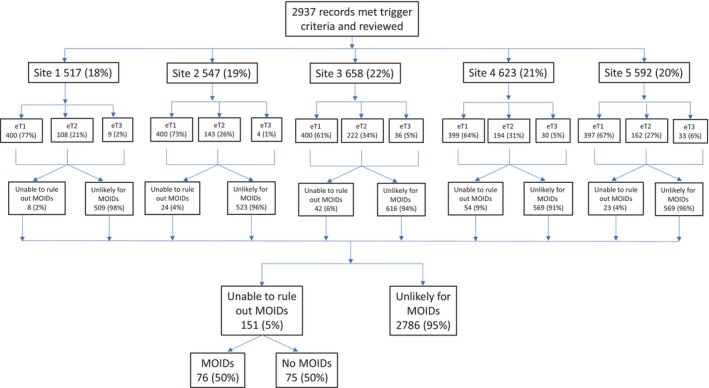
Charts reviewed across participating sites. eT1, electronic Trigger 1 (unscheduled return visit with admission within 10 days); eT2, electronic Trigger 2 (care escalation to ICU within 24 h after inpatient admission); eT3, electronic Trigger 3 (death within 24 h of ED arrival or admission).

We identified 2937 cases across five pediatric ED sites that met trigger criteria (Trigger 1 1996 [68%], Trigger 2 829 [28%], Trigger 3 112 [4%; six erroneous duplicate patients were identified as a limitation of implementing the data query at a participating site and were removed during data analysis so that the patient was counted as a single event]). Reviewers excluded 2786 (95%) records as very unlikely for MOIDs (see Data S5 for reason excluded breakdown). In the remaining 151 (5%) records, we applied the Revised Safer Dx Instrument to identify MOIDs. Of these records, 76 had a MOID (50.3%). Figure 1 displays a consort diagram of charts reviewed across participating sites. The overall frequency of MOIDs was 2.6% (76/2937) for the entire cohort, 3.0% (60/1996) for Trigger 1, 1.9% (16/829) for Trigger 2, and 0% (0/112) for Trigger 3. The proportion of charts with MOIDs ranged from 0.6% to 4.4% across sites. Six cases suggestive of a MOID required scoring and discussion between both site reviewers. No cases reviewed required final adjudication by the investigative team.

Table 1 describes the entire study population stratified by trigger. As some Trigger 1 records had multiple return visits attached to a single index visit, resulting in duplicated index visit rows; return visits (*n* = 1996) are examined separately to preserve the true number of index visits (*n* = 1981) without losing data for returns. Table 2 displays demographics of return visits. Specific to Trigger 3, a greater proportion of patients were younger than 1 year old (33.0%) and Black (47.3%). Most patients for Triggers 1 and 2 had a reason for visit associated with the NHAMCS module symptom, while the greatest frequency of patients for Trigger 3 had a reason for visit associated with the module injuries and adverse effects.

**TABLE 1:** Study population demographics, unique visits by trigger.

	All triggers (*N* = 2919)	Trigger 1^a^ (*n* = 1981)	Trigger 2^a^ (*n* = 829)	Trigger 3^a^ (*n* = 112)
Sex				
Female	1386 (47.5)	967 (48.8)	370 (44.6)	52 (46.8)
Male	1533 (52.5)	1014 (51.2)	459 (55.4)	60 (53.2)
Age (years)	6.43 (±6.82)	6.76 (±6.74)	5.69 (±6.81)	6.04 (±7.94)
<1	620 (21.2)	353 (17.8)	230 (27.7)	37 (33.0)
1–5	1086 (37.2)	755 (38.1)	298 (35.9)	34 (30.4)
6–15	752 (25.8)	534 (27.0)	191 (23.0)	27 (24.1)
>15	461 (15.8)	339 (17.1)	110 (13.3)	14 (12.5)
Race				
American Indian or Alaska Native	5 (0.2)	1 (0.1)	4 (0.5)	0
Asian	76 (2.6)	53 (2.7)	23 (2.8)	0
Black	940 (32.2)	654 (33.0)	235 (28.3)	53 (47.3)
Multiple	13 (0.5)	9 (0.5)	3 (0.4)	1 (0.9)
Native Hawaiian or Pacific Islander	5 (0.2)	3 (0.2)	2 (0.2)	0
Other	500 (17.1)	349 (17.6)	135 (16.3)	16 (14.3)
White	1321 (45.3)	885 (44.7)	408 (49.2)	29 (25.9)
Missing	59 (2.0)	27 (1.4)	19 (2.3)	13 (11.6)
Ethnicity				
Hispanic/Latino	513 (17.6)	347 (17.5)	154 (18.6)	12 (10.7)
Not Hispanic/Latino	2388 (81.8)	1631 (82.3)	667 (80.5)	93 (83.0)
Not reported/missing	18 (0.6)	3 (0.2)	8 (1.0)	7 (6.2)
Insurance provider				
Medicaid	1648 (56.5)	1148 (58.0)	432 (52.1)	71 (63.4)
Medicare	7 (0.2)	3 (0.2)	3 (0.4)	1 (0.9)
Other	13 (0.4)	8 (0.4)	5 (0.6)	0
Other (governmental)	8 (0.3)	6 (0.3)	2 (0.2)	0
Private	1191 (40.8)	789 (39.8)	375 (45.2)	27 (24.1)
Self‐pay	37 (1.3)	21 (1.1)	9 (1.1)	7 (6.2)
Missing	15 (0.5)	6 (0.3)	3 (0.4)	6 (5.4)
Visit arrival month				
January	265 (9.1)	170 (8.6)	88 (10.6)	7 (6.2)
February	224 (7.7)	150 (7.6)	63 (7.6)	11 (9.8)
March	270 (9.3)	204 (10.3)	61 (7.4)	5 (4.5)
April	233 (8.0)	152 (7.7)	71 (8.6)	10 (8.9)
May	239 (8.2)	162 (8.2)	64 (7.7)	13 (11.6)
June	182 (6.2)	141 (7.1)	35 (4.2)	7 (6.2)
July	195 (6.7)	124 (6.3)	59 (7.1)	12 (10.7)
August	209 (7.2)	147 (7.4)	52 (6.3)	10 (8.9)
September	222 (7.6)	154 (7.8)	60 (7.2)	8 (7.1)
October	252 (8.7)	172 (8.7)	71 (8.6)	10 (8.9)
November	287 (9.8)	183 (9.2)	92 (11.1)	13 (11.6)
December	341 (11.7)	222 (11.2)	113 (13.6)	6 (5.4)
Visit arrival day				
Monday	476 (16.3)	338 (17.1)	121 (14.6)	17 (15.2)
Tuesday	455 (15.6)	301 (15.2)	140 (16.9)	14 (12.5)
Wednesday	440 (15.1)	310 (15.6)	119 (14.4)	11 (9.8)
Thursday	408 (14.0)	266 (13.4)	129 (15.6)	15 (13.4)
Friday	375 (12.9)	247 (12.5)	111 (13.4)	17 (15.2)
Saturday	377 (12.9)	264 (13.3)	94 (11.3)	19 (17.0)
Sunday	388 (13.3)	255 (12.9)	115 (13.9)	19 (17.0)
Time of visit				
12:01 a.m.–7:59 a.m.	488 (16.7)	313 (15.8)	146 (17.6)	31 (27.7)
8:00 a.m.–4:00 p.m.	1304 (44.7)	874 (44.1)	378 (45.6)	52 (46.4)
4:01 p.m.–12:00 a.m.	1127 (38.6)	794 (40.1)	305 (36.8)	29 (25.9)
ED length of stay (h)^b^				
Median (IQR)	4.8 (3.20–6.69)	4.47 (3.00–6.28)	5.67 (4.25–7.60)	4.10 (2.24–5.38)
Reason for visit module				
Administrative	6 (0.2)	0	3 (0.4)	3 (2.7)
Diagnostic, screening, and preventive	65 (2.2)	62 (3.1)	3 (0.4)	0
Disease	233 (8.0)	165 (8.3)	63 (7.6)	6 (5.4)
Injuries and adverse effects	216 (7.4)	130 (6.6)	44 (5.3)	43 (38.4)
Symptom	2158 (73.9)	1467 (74.1)	671 (80.9)	21 (18.8)
Test results	16 (0.6)	14 (0.7)	2 (0.2)	0
Treatment	28 (1.0)	22 (1.1)	6 (0.7)	0
Uncodable entries^c^	197 (6.8)	121 (6.1)	37 (4.5)	39 (34.8)
Final first diagnosis system category				
BLD	150 (5.1)	126 (6.4)	21 (2.5)	3 (2.7)
CIR	107 (3.7)	37 (1.9)	24 (2.9)	46 (41.1)
DIG	225 (7.7)	188 (9.5)	35 (4.2)	2 (1.8)
EAR	46 (1.6)	43 (2.2)	3 (0.4)	0 (0)
END	122 (4.2)	72 (3.6)	50 (6)	1 (0.9)
EXT				
EYE	25 (0.9)	21 (1.1)	4 (0.5)	0 (0)
FAC	17 (0.6)	12 (0.6)	2 (0.2)	3 (2.7)
GEN	60 (2.1)	51 (2.6)	9 (1.1)	0 (0)
INF	178 (6.1)	92 (4.6)	73 (8.8)	13 (11.6)
INJ	183 (6.3)	114 (5.8)	55 (6.6)	15 (13.4)
MAL	26 (0.9)	14 (0.7)	12 (1.5)	0 (0)
MBD	59 (2)	55 (2.8)	5 (0.6)	0 (0)
MUS	38 (1.3)	38 (1.9)	0 (0)	0 (0)
NEO	68 (2.3)	48 (2.4)	15 (1.8)	5 (4.5)
NVS	192 (6.6)	137 (6.9)	45 (5.4)	10 (8.9)
PNL	61 (2.1)	39 (2)	16 (1.9)	6 (5.4)
PRG	1 (0)	1 (0.1)	0 (0)	0 (0)
RSP	851 (29.2)	444 (22.4)	404 (48.7)	3 (2.7)
SKN	61 (2.1)	57 (2.9)	4 (0.5)	0 (0)
SYM	357 (12.2)	344 (17.4)	11 (1.3)	2 (1.8)
XXX	74 (2.5)	42 (2.1)	31 (3.7)	1 (0.9)
Missing	18 (0.6)	6 (0.3)	10 (1.2)	2 (1.8)

*Note*: Data are reported as *n* (%) or mean (±SD) unless otherwise specified. See User Guide for the Clinical Classifications Software Refined (CCSR) for ICD‐10‐CM diagnoses Table 1 for complete descriptions.

Abbreviations: BLD, diseases of the blood and blood‐forming organs and certain disorders involving the immune mechanism; CIR, diseases of the circulatory system; DIG, diseases of the digestive system; EAR, diseases of the ear and mastoid process; END, endocrine, nutritional, and metabolic diseases; EXT, external causes of morbidity; EYE, diseases of the eye and adnexa; FAC, factors influencing health status and contact with health services; GEN, diseases of the genitourinary system; INF, certain infectious and parasitic diseases; INJ, injury, poisoning, and certain other consequences of external causes; MAL, congenital malformations, deformations, and chromosomal abnormalities; MBD, mental, behavioral, and neurodevelopmental disorders; MUS, diseases of the musculoskeletal system and connective tissue; NEO, neoplasms; NVS, diseases of the nervous system; PNL, certain conditions originating in the perinatal period; PRG, pregnancy, childbirth, and the puerperium; RSP, diseases of the respiratory system; SKN, diseases of the skin and subcutaneous tissue; SYM, symptoms, signs, and abnormal clinical and laboratory findings, not elsewhere classified; XXX, codes identified as an unacceptable first‐listed diagnosis.

^a^ Individual trigger columns do not equal all triggers total due to overlap in records between triggers.

^b^ Total *n* available for 2724 records, 667 for Trigger 2, and 79 for Trigger 3.

^c^ Uncodable entries: Can include blank/missing/otherwise categorized uncodable.

**TABLE 2:** Study population demographics, Trigger 1 return visits.

	Trigger 1 return visits (*n* = 1996)
Visit arrival month	
January	160 (8.0)
February	152 (7.6)
March	203 (10.2)
April	160 (8.0)
May	163 (8.2)
June	145 (7.3)
July	125 (6.3)
August	151 (7.6)
September	151 (7.6)
October	170 (8.5)
November	183 (9.2)
December	233 (11.7)
Visit arrival day	
Monday	340 (17.0)
Tuesday	276 (13.8)
Wednesday	308 (15.4)
Thursday	312 (15.6)
Friday	273 (13.7)
Saturday	234 (11.7)
Sunday	253 (12.7)
Time of visit	
12:01 a.m.–7:59 a.m.	332 (16.6)
8:00 a.m.–4:00 p.m.	923 (46.2)
4:01 pm–12:00 am	741 (37.1)
ED length of stay (h)	
Median (IQR)	5.9 (4.00–8.40)
Reason for visit module	
Administrative	0
Diagnostic, screening, and preventive	2 (0.1)
Disease	197 (9.9)
Injuries and adverse effects	84 (4.2)
Symptom	1372 (68.7)
Test results	45 (2.3)
Treatment	45 (2.3)
Uncodable entries^a^	251 (12.6)
Final first diagnosis system category	
BLD	145 (7.3)
CIR	24 (1.2)
DIG	251 (12.7)
EAR	18 (0.9)
END	165 (8.3)
EXT	0
EYE	18 (0.9)
FAC	6 (0.3)
GEN	48 (2.4)
INF	96 (4.9)
INJ	93 (4.7)
MAL	13 (0.7)
MBD	91 (4.6)
MUS	27 (1.4)
NEO	56 (2.8)
NVS	146 (7.4)
PNL	37 (1.9)
PRG	2 (0.1)
RSP	486 (24.5)
SKN	71 (3.6)
SYM	131 (6.6)
XXX	62 (3.1)
Missing	8 (0.4)

*Note*: Data are reported as *n* (%) unless otherwise specified. See user guide for the Clinical Classifications Software Refined (CCSR) for ICD‐10‐CM diagnoses Table 1 for complete descriptions.

Abbreviations: BLD, diseases of the blood and blood‐forming organs and certain disorders involving the immune mechanism; CIR, diseases of the circulatory system; DIG, diseases of the digestive system; EAR, diseases of the ear and mastoid process; END, endocrine, nutritional, and metabolic diseases; EXT, external causes of morbidity; EYE, diseases of the eye and adnexa; FAC, factors influencing health status and contact with health services; GEN, diseases of the genitourinary system; INF, certain infectious and parasitic diseases; INJ, injury, poisoning, and certain other consequences of external causes; MAL, congenital malformations, deformations, and chromosomal abnormalities; MBD, mental, behavioral, and neurodevelopmental disorders; MUS, diseases of the musculoskeletal system and connective tissue; NEO, neoplasms; NVS, diseases of the nervous system; PNL, certain conditions originating in the perinatal period; PRG, pregnancy, childbirth, and the puerperium; RSP, diseases of the respiratory system; SKN, diseases of the skin and subcutaneous tissue; SYM, symptoms, signs and abnormal clinical and laboratory findings, not elsewhere classified; XXX, codes identified as an unacceptable first‐listed diagnosis.

^a^ Uncodable entries line: Can include blank/missing/otherwise categorized uncodable.

To discern possible factors associated with a MOID, we analyzed records by trigger and overall scoring by the Revised Safer Dx Instrument (status determined as Safer Dx+ for records scored as >4 on the summative item 13 and Safer Dx− for records scored as <4). No Trigger 3 records reviewed using the Revised Safer Dx Instrument were suggestive of a MOID. Complete descriptive statistics are provided in Table 3. A greater proportion of patients ultimately assessed as likely to have a MOID were Hispanic (27.6%, 10.7%) and covered by Medicaid insurance (65.8%, 45.3%) and had a shorter index ED length of stay (4.03 h, 6.00 h) than patients ultimately assessed as unlikely to have a MOID. Related to Trigger 1, a greater proportion of cases suggestive of a MOID had a shorter index ED visit length of stay than return ED visit length of stay (4.00 h, 6.70 h). Fewer Trigger 1 return visits were categorized under the symptoms, signs, and abnormal clinical and laboratory findings, not elsewhere classified final first diagnosis system. Related to Trigger 2, a greater proportion of cases suggestive of a MOID arrived to the ED from 4:01 p.m.–7:59 a.m. (68.7%).

**TABLE 3:** Study population demographics by Safer Dx status (status determined as Safer Dx+ for records scored as >4 on the summative item 13 and Safer Dx− for records scored as <4; no Trigger 3 records were Safer Dx+).

	All triggers (Safer Dx total reviewed+), *N* = 76	All triggers (Safer Dx total reviewed−), *N* = 75	*p‐*value	Trigger 1 (Safer Dx+), *n* = 60 (%)	Trigger 1 (Safer Dx−), *n* = 43 (%)	*p‐*value	Trigger 2 (Safer Dx+), *n* = 16	Trigger 2 (Safer Dx−), *n* = 28 (%)	*p‐*value	Trigger 3 (Safer Dx−), *n* = 5 (%)
Sex			1			0.83			0.93	
Female	40 (52.6)	39 (52.0)		31 (51.7)	24 (55.8)		9 (56.2)	14 (50.0)		2 (40.0)
Male	36 (47.4)	36 (48.0)		29 (48.3)	19 (44.2)		7 (43.8)	14 (50.0)		3 (60.0)
Age (years)			0.1			0.55		8.93 (7.97)	0.06	5.20 (6.83)
Mean (±SD)	6.95 (±5.85)	8.43 (±7.00)	6	7.57 (±5.90)	8.30 (±6.40)		4.62 (±5.16)			
			0.11			0.39			0.25	
<1	8 (10.5)	9 (12.0)		4 (6.7)	4 (9.3)		4 (25.0)	4 (14.3)		1 (20.0)
1–5	32 (42.1)	25 (33.3)		24 (40.0)	15 (34.9)		8 (50.0)	9 (32.1)		2 (40.0)
6–15	28 (36.8)	22 (29.3)		25 (41.7)	14 (32.6)		3 (18.8)	7 (25.0)		1 (20.0)
>15	8 (10.5)	19 (25.3)		7 (11.7)	10 (23.3)		1 (6.2)	8 (28.6)		1 (20.0)
Race			0.12			0.19			0.16	
American Indian or Alaska Native	0	0		0	0		0	0		0
Asian	3 (3.9)	2 (2.7)		2 (3.3)	2 (4.7)		1 (6.2)	0		0
Black	23 (30.3)	28 (37.3)		17 (28.3)	18 (41.9)		6 (37.5)	8 (28.6)		3 (60.0)
Multiple	0 (0.0)	1 (1.3)		0	1 (2.3)		0	0		0
Native Hawaiian or Pacific Islander	0	0		0	0		0	0		0
Other	16 (21.1)	5 (6.7)		14 (23.3)	3 (7.0)		2 (12.5)	1 (3.6)		1 (20.0)
White	30 (39.5)	37 (49.3)		24 (40.0)	18 (41.9)		6 (37.5)	19 (67.9)		0
Missing	4 (5.3)	2 (2.7)		3 (5.0)	1 (2.3)		1 (6.2)	0		1 (20.0)
Ethnicity			0.03			0.08			0.17	
Hispanic/Latino	21 (27.6)	8 (10.7)		17 (28.3)	5 (11.6)		4 (25.0)	3 (10.7)		0
Not Hispanic/Latino	54 (71.1)	66 (88.0)		43 (71.7)	38 (88.4)		11 (68.8)	25 (89.3)		4 (80.0)
Missing	1 (1.3)	1 (1.3)		0	0		1 (6.2)	0		1 (20.0)
Insurance provider			0.04			0.09			0.11	
Medicaid	50 (65.8)	34 (45.3)		38 (63.3)	19 (44.2)		12 (75.0)	12 (42.9)		4 (80.0)
Medicare	0 (0.0)	1 (1.3)		0	0		0	1 (3.6)		0
Other	0 (0.0)	0		0	0		0	0		0
Other (governmental)	0 (0.0)	0		0	0		0	0		0
Private	25 (32.9)	40 (53.3)		21 (35.0)	24 (55.8)		4 (25.0)	15 (53.6)		1 (20.0)
Self‐pay	1 (1.3)	0 (0.0)		1 (1.7)	0		0	0		0
Visit arrival month			0.21			0.47			0.38	
January	3 (3.9)	9 (12.0)		2 (3.3)	4 (9.3)		1 (6.2)	4 (14.3)		1 (20.0)
February	5 (6.6)	5 (6.7)		4 (6.7)	2 (4.7)		1 (6.2)	2 (7.1)		1 (20.0)
March	10 (13.2)	5 (6.7)		9 (15.0)	4 (9.3)		1 (6.2)	1 (3.6)		0
April	3 (3.9)	7 (9.3)		3 (5.0)	4 (9.3)		0	3 (10.7)		0
May	10 (13.2)	2 (2.7)		6 (10.0)	1 (2.3)		4 (25.0)	1 (3.6)		0
June	7 (9.2)	5 (6.7)		7 (11.7)	4 (9.3)		0	1 (3.6)		0
July	6 (7.9)	3 (4.0)		5 (8.3)	1 (2.3)		1 (6.2)	2 (7.1)		0
August	8 (10.5)	9 (12.0)		6 (10.0)	6 (14.0)		2 (12.5)	3 (10.7)		0
September	4 (5.3)	8 (10.7)		2 (3.3)	5 (11.6)		2 (12.5)	3 (10.7)		0
October	10 (13.2)	11 (14.7)		9 (15.0)	5 (11.6)		1 (6.2)	6 (21.4)		1 (20.0)
November	4 (5.3)	5 (6.7)		4 (6.7)	3 (7.0)		0	1 (3.6)		1 (20.0)
December	6 (7.9)	6 (8.0)		3 (5.0)	4 (9.3)		3 (18.8)	1 (3.6)		1 (20.0)
Visit arrival month (Trigger 1 return)						0.53				
January				1 (1.7)	4 (9.3)					
February				5 (8.3)	2 (4.7)					
March				8 (13.3)	4 (9.3)					
April				3 (5.0)	3 (7.0)					
May				7 (11.7)	2 (4.7)					
June				6 (10.0)	3 (7.0)					
July				6 (10.0)	2 (4.7)					
August				6 (10.0)	5 (11.6)					
September				2 (3.3)	5 (11.6)					
October				8 (13.3)	5 (11.6)					
November				5 (8.3)	4 (9.3)					
December				3 (5.0)	4 (9.3)					
Visit arrival day			0.40			0.26			0.45	
Monday	13 (17.1)	11 (14.7)		11 (18.3)	9 (20.9)		2 (12.5)	2 (7.1)		0
Tuesday	9 (11.8)	5 (6.7)		6 (10.0)	4 (9.3)		3 (18.8)	1 (3.6)		0
Wednesday	18 (23.7)	15 (20.0)		17 (28.3)	11 (25.6)		1 (6.2)	4 (14.3)		0
Thursday	6 (7.9)	16 (21.3)		3 (5.0)	8 (18.6)		3 (18.8)	7 (25.0)		2 (40.0)
Friday	8 (10.5)	8 (10.7)		5 (8.3)	5 (11.6)		3 (18.8)	2 (7.1)		1 (20.0)
Saturday	12 (15.8)	11 (14.7)		10 (16.7)	4 (9.3)		2 (12.5)	6 (21.4)		1 (20.0)
Sunday	10 (13.2)	9 (12.0)		8 (13.3)	2 (4.7)		2 (12.5)	6 (21.4)		1 (20.0)
Visit arrival day (Trigger 1 return)						0.78				
Monday				7 (11.7)	6 (14.0)					
Tuesday				7 (11.7)	4 (9.3)					
Wednesday				11 (18.3)	7 (16.3)					
Thursday				12 (20.0)	6 (14.0)					
Friday				9 (15.0)	12 (27.9)					
Saturday				6 (10.0)	4 (9.3)					
Sunday				8 (13.3)	4 (9.3)					
Time of visit			0.39			0.93			0.02	
12:01 a.m.–7:59 a.m.	15 (19.7)	9 (12.0)		10 (16.7)	6 (14.0)		5 (31.2)	1 (3.6)		2 (40.0)
8:00 a.m.–4:00 p.m.	31 (40.8)	36 (48.0)		26 (43.3)	19 (44.2)		5 (31.2)	17 (60.7)		0
4:01 p.m.‐12:00 a.m.	30 (39.5)	30 (40.0)		24 (40.0)	18 (41.9)		6 (37.5)	10 (35.7)		3 (60.0)
Time of visit (Trigger 1 return)						0.87				
12:01 a.m.–7:59 a.m.				4 (6.7)	4 (9.3)					
8:00 a.m.–4:00 p.m.				34 (56.7)	23 (53.5)					
4:01 p.m.–12:00 a.m.				22 (36.7)	16 (37.2)					
ED length of stay (h)			0.01			0.01		7.50 (4.73–11.25)	0.41	6.51 (±5.46)
Median (IQR); mean (±SD)	4.03 (2.90–5.56)	6.00 (4.12–8.14)		4.00 (2.77–5.03]	5.10 (3.62–7.38)		5.20 (4.05–7.28)			
ED length of stay (h) (Trigger 1 return)						0.56				
Median (IQR)				6.70 [4.50– 8.55)	6.75 (5.13–9.07)					
Reason for visit module			0.55			0.31			0.43	
Administrative	0	0		0	0		0	0		0
Diagnostic, screening, and preventive	1 (1.3)	1 (1.3)		1 (1.7)	1 (2.3)		0	0		0
Disease	2 (2.6)	3 (4.0)		0	2 (4.7)		2 (12.5)	1 (3.6)		0
Injuries and adverse effects	3 (3.9)	8 (10.7)		2 (3.3)	4 (9.3)		1 (6.2)	3 (10.7)		1 (20.0)
Symptom	65 (85.5)	57 (76.0)		53 (88.3)	34 (79.1)		12 (75.0)	23 (82.1)		1 (20.0)
Test results	0	0		0	0		0	0		0
Treatment	0	1 (1.3)		0	0		0	1 (3.6)		0
Uncodable entries^a^	5 (6.6)	5 (6.7)		4 (6.7)	2 (4.7)		1 (6.2)	0 (0.0)		3 (60.0)
Reason for visit module (Trigger 1 return)						0.51				
Administrative				0	0					
Diagnostic, screening, and preventive				0	0					
Disease				2 (3.3)	4 (9.3)					
Injuries and adverse effects				4 (6.7)	5 (11.6)					
Symptom				44 (73.3)	28 (65.1)					
Test results				2 (3.3)	1 (2.3)					
Treatment				0	1 (2.3)					
Uncodable entries^a^				8 (13.3)	4 (9.3)					
Final first diagnosis system category			0.62			0.68			0.23	
BLD	3 (3.9)	2 (2.7)		1 (1.7)	2 (4.7)		2 (12.5)	0 (0.0)		0
CIR	1 (1.3)	4 (5.3)		1 (1.7)	0 (0.0)		0 (0.0)	3 (10.7)		1 (20.0)
DIG	7 (9.2)	9 (12.0)		7 (11.7)	7 (16.3)		0 (0.0)	2 (7.1)		0
EAR	4 (5.3)	0 (0.0)		4 (6.7)	0 (0.0)		0	0		0
END	3 (3.9)	3 (4.0)		2 (3.3)	2 (4.7)		1 (6.2)	1 (3.6)		1 (20.0)
EXT	0	0		0	0		0	0		0
EYE	0 (0.0)	1 (1.3)		0	0		0 (0.0)	1 (3.6)		0
FAC	0 (0.0)	1 (1.3)		0 (0.0)	1 (2.3)		0	0		0
GEN	3 (3.9)	3 (4.0)		3 (5.0)	3 (7.0)		0	0		0
INF	6 (7.9)	5 (6.7)		2 (3.3)	2 (4.7)		4 (25.0)	2 (7.1)		1 (20.0)
INJ	5 (6.6)	4 (5.3)		3 (5.0)	2 (4.7)		2 (12.5)	1 (3.6)		1 (20.0)
MAL	0	0		0	0		0	0		0
MBD	1 (1.3)	1 (1.3)		1 (1.7)	0 (0.0)		0 (0.0)	1 (3.6)		0
MUS	5 (6.6)	2 (2.7)		5 (8.3)	2 (4.7)		0	0		0
NEO	2 (2.6)	2 (2.7)		2 (3.3)	0 (0.0)		0 (0.0)	2 (7.1)		0
NVS	8 (10.5)	4 (5.3)		5 (8.3)	1 (2.3)		3 (18.8)	3 (10.7)		0
PNL	0 (0.0)	1 (1.3)		0 (0.0)	1 (2.3)		0	0		0
PRG	0	0		0	0		0	0		0
RSP	13 (17.1)	18 (24.0)		9 (15.0)	8 (18.6)		4 (25.0)	10 (35.7)		0
SKN	1 (1.3)	2 (2.7)		1 (1.7)	1 (2.3)		0 (0.0)	1 (3.6)		0
SYM	14 (18.4)	11 (14.7)		14 (23.3)	10 (23.3)		0	0		1 (20.0)
XXX	0 (0.0)	2 (2.7)		0 (0.0)	1 (2.3)		0 (0.0)	1 (3.6)		0
Final first diagnosis system category (Trigger 1 return)						0.44				
BLD				2 (3.3)	2 (4.7)					
CIR				1 (1.7)	2 (4.7)					
DIG				10 (16.7)	6 (14.0)					
EAR				0	1 (2.3)					
END				3 (5.0)	4 (9.3)					
EXT				0	0					
EYE				1 (1.7)	0					
FAC				0	0					
GEN				2 (3.3)	4 (9.3)					
INF				2 (3.3)	2 (4.7)					
INJ				5 (8.3)	4 (9.3)					
MAL				0	0					
MBD				0	1 (2.3)					
MUS				5 (8.3)	3 (7.0)					
NEO				5 (8.3)	2 (4.7)					
NVS				5 (8.3)	2 (4.7)					
PNL				0	1 (2.3)					
PRG				0	0					
RSP				14 (23.3)	6 (14.0)					
SKN				0	3 (7.0)					
SYM				2 (3.3)	0					
XXX				2 (3.3)	0					
Missing				1 (1.7)	0					
Final Safer Dx score								N/A		N/A
Mean (±SD)	5.29 (±0.64)	N/A		5.28 (±0.64)	N/A		5.44 (±0.81)			
Harm assessment										
No harm	3 (3.9)	N/A		3 (5.0)	N/A		0	N/A		N/A
Error, no harm	32 (42.1)	N/A		28 (46.7)	N/A		4 (25.0)	N/A		N/A
Error, harm	41 (53.9)	N/A		29 (48.3)	N/A		12 (75.0)	N/A		N/A
Error, death	0	N/A		0	N/A		0	N/A		N/A

*Note*: See User Guide for the Clinical Classifications Software Refined (CCSR) for ICD‐10‐CM diagnoses Table 1 for complete descriptions.

Abbreviations: BLD, diseases of the blood and blood‐forming organs and certain disorders involving the immune mechanism; CIR, diseases of the circulatory system; DIG, diseases of the digestive system; EAR, diseases of the ear and mastoid process; END, endocrine, nutritional, and metabolic diseases; EXT, external causes of morbidity; EYE, diseases of the eye and adnexa; FAC, factors influencing health status and contact with health services; GEN, diseases of the genitourinary system; INF, certain infectious and parasitic diseases; INJ, injury, poisoning, and certain other consequences of external causes; MAL, congenital malformations, deformations, and chromosomal abnormalities; MBD, mental, behavioral, and neurodevelopmental disorders; MUS, diseases of the musculoskeletal system and connective tissue; NEO, neoplasms; NVS, diseases of the nervous system; PNL, certain conditions originating in the perinatal period; PRG, pregnancy, childbirth, and the puerperium; RSP, diseases of the respiratory system; SKN, diseases of the skin and subcutaneous tissue; SYM, symptoms, signs, and abnormal clinical and laboratory findings, not elsewhere classified; XXX, codes identified as an unacceptable first‐listed diagnosis.

^a^ Uncodable entries line: Can include blank/missing/otherwise categorized uncodable.

We describe the reviewer‐determined missed diagnosis and level of harm for the 76 cases suggestive of a MOID in Table 4. The most common missed diagnoses in this cohort were related to the nervous system (19%, 25%), gastrointestinal system (17%, 22.4%), pulmonary complaints (13%, 17.1%), or kidney complaints (10%, 13.2%). Brain lesions, infections, or hemorrhage (14%, 18.4%), pneumonias and lung abscess (12%, 15.8%), and appendicitis (8%, 10.5%) were the top three missed diagnoses overall, as well as for Trigger 1 (10%, 16.7%; 9%, 15%; and 7%, 11.7%, respectively). The top three missed diagnoses for Trigger 2 were brain lesions, infections, or hemorrhage (4%, 25%), pneumonias and lung abscess (3%, 18.8%), and infections (2%, 12.5%).

**TABLE 4:** Missed diagnoses and level of harm^a^ by disease system.

Trigger 1 (*n* = 60)		Trigger 2 (*n* = 16)	
**Gastrointestinal**	**16**	**Nervous system**	**6**
Appendicitis	7	Brain lesions/infections/hemorrhage^b^	4
Error, harm	5	Error, harm	3
Error, no harm	2	Error, no harm	1
Cholecystitis	1	Infant botulism	1
Error, no harm	1	Error, no harm	1
Constipation	1	Meningitis	1
No harm	1	Error, harm	1
Diaphragmatic hernia	1	**Pulmonary**	**4**
Error, harm	1	Acute chest	1
Esophagitis/gastritis	1	Error, harm	1
Error, harm	1	Pneumonias and lung abscess	3
Intussusception	1	Error, harm	2
Error, harm	1	Error, no harm	1
Pancreatitis	2	**Other**	**3**
Error, no harm	2	Infections^c^	2
Pinworms	1	Error, harm	2
Error, no harm	1	Kawasaki disease	1
Pyloric stenosis	1	Error, harm	1
Error, no harm	1	**Gastrointestinal**	**1**
**Nervous system**	**13**	Appendicitis	1
Brain lesions/infections/hemorrhage^b^	10	Error, harm	1
Error, harm	6	**Ear, nose, throat**	**1**
Error, no harm	4	Vocal cord dysfunction	1
Infantile seizures	2	Error, no harm	1
Error, harm	1	**Kidney**	**1**
Error, no harm	1	Pyelonephritis/urinary tract infection	1
Meningitis	1	Error, harm	1
Error, no harm	1		
**Pulmonary**	**9**		
Pneumonias and lung abscess	9		
Error, harm	3		
Error, no harm	6		
**Kidney**	**9**		
Hemolytic uremic syndrome	1		
Error, harm	1		
Nephroblastoma	1		
Error, no harm	1		
Nephrotic syndrome	1		
Error, no harm	1		
Pyelonephritis/urinary tract infection	5		
Error, harm	5		
Wilms tumor	1		
Error, no harm	1		
**Bone**	**5**		
Osteomyelitis	5		
Error, harm	3		
Error, no harm	1		
No harm	1		
**Ear, nose and throat**	**4**		
Pansinusitis with orbital cellulitis	1		
Error, no harm	1		
Retropharyngeal abscess	1		
Error, harm	1		
Septal hematoma	1		
Error, no harm	1		
Tracheitis	1		
Error, no harm	1		
**Other**	**4**		
Hypoglycemia	1		
Error, harm	1		
Infections^c^	2		
Error, no harm	1		
No harm	1		
Severe iron deficiency anemia	1		
Error, no harm	1		

*Note*: We had bolded the disease system main group rows to help those stand out.

^a^ Level of harm includes: *no harm* (Category A—circumstances or events that have the capacity to cause error); *error, no harm* (Category B—an error occurred but the error did not reach the patient (an “error of omission” does reach the patient); Category C—an error occurred that reached the patient but did not cause patient harm; or Category D—an error occurred that reached the patient and required monitoring to confirm that it resulted in no harm to the patient and/or required intervention to preclude harm); *error, harm* (Category E—an error occurred that may have contributed to or resulted in temporary harm to the patient and required intervention; Category F—an error occurred that may have contributed to or resulted in temporary harm to the patient and required initial or prolonged hospitalization; or Category G—an error occurred that may have contributed to or resulted in permanent patient harm).

^b^ Trigger 1 includes one empyema, one encephalitis, two epidural/subdural abscesses, two epidural/subdural hematomas (one due to nonaccidental trauma), one increased intracranial pressure, one ventriculomegaly, two tumor related (medulloblastoma, metastatic lesion). Trigger 2 includes one empyema, one encephalitis, one epidural/subdural hematomas (due to nonaccidental trauma), one VP shunt infection.

^c^ Trigger 1 includes one influenza, one transverse myelitis. Trigger 2 includes one influenza, one MSSA sepsis.

Reviewers indicated the majority of MOIDs cases resulted in patient harm (41/76, 53.9%). By trigger, 48.3% of Trigger 1 cases resulted in harm (29/60) and 75% of Trigger 2 cases resulted in harm (12/16). Across the two triggers, top missed diagnoses resulting in harm were brain lesions, infections, or hemorrhage (9/41, 22%); appendicitis (6/41, 14.6%); pyelonephritis or urinary tract infections (6/41, 14.6%); and pneumonias and lung abscess (5/41, 12.2%). Full details related to MOIDs cases are available in Data S6. Of all cases suggestive of a MOID, the ultimate correct diagnosis was documented in the differential diagnosis list at the initial ED visit for 23/76 cases (30.3%). The ultimate correct diagnosis was not documented in a differential list for 34/76 cases (44.7%) and no differential list was documented for 19/76 cases (25%). Many MOID cases had index reason for visit of fever (14/76, 18.4%), stomach and abdominal pain, cramps and spasms (9/76, 11.4%), and labored or difficult breathing (8/76, 10.5%). Contributing factor groups were able to be assigned in most cases (50/76, 65.8%). Reviewers determined patient–provider factors contributed in 52.6% (40/76) of MOID cases, followed by patient factors 21.1% (16/76), system factors 13.2% (10/76), and provider factors 10.5% (8/76). A majority of MOID cases were discovered due to a failure of original symptoms or signs to resolve (attributed to 45/76 cases, 59.2%). Other factors that impacted MOID discovery were evolution of the original symptoms or signs (attributed to 35/76 cases, 46.1%), unplanned return visits (attributed to 29/76 cases, 38.2%), and the availability of new data (attributed to 26/76 cases, 34.2%). MOIDs were unlikely to be discovered as part of planned follow‐up (attributed to six of 76 cases, 7.9%) or in the hospital quality assurance process (attributed to one of 76 cases, 1.3%).

We analyzed cases reviewed using the Revised Safer Dx Instrument with logistic regression (Table 5). Univariate analyses indicated that, compared to patients of <1 year of age, patients between the ages of 1 and 5 (odds ratio [OR] 2.3, 95% confidence interval [CI] 1.1–5.4) and those between the ages of 6 and 15 (OR 3.0, [95% CI 1.4–7]) had greater odds of being classified as suggestive of a MOID. Interestingly, patients aged 15 years and older were not more likely than patients <1 year of age to be classified as suggestive of a MOID, indicating that the association of age with the odds of being likely for a MOID may be nonlinear and potentially U‐shaped. The association between length of stay and patients being classified as likely for a MOID was not borne out in the full sample (OR 0.94, 95% CI 0.86–1.006). A multivariable regression of length of stay and patient age on classification of being likely for a MOID showed little change from the univariate analyses, with patients aged 1–5 years (OR 2.34, 95% CI 1.12–5.50) and 6–15 years (OR 3.16, 95% CI 1.49–7.49) having greater odds of being classified as likely for a MOID than patients aged <1 year and length of stay not correlating with patients being classified as likely for a MOID (OR 0.94, 95% CI 0.86–1.003).

**TABLE 5:** Logistic regression models.

Univariate logistic regression association with MOID				
	OR	CI low	CI high	*p*‐value
Age (ref <1 year)				
Age: 1–5	2.323	1.117	5.445	0.034
Age: 15+	1.351	0.494	3.698	0.55
Age: 6–15	2.959	1.402	7	0.007
Age (continuous; years)				
Age	1.011	0.978	1.044	0.499
Sex (ref “male”)				
Female	1.235	0.782	1.956	0.365
Race (ref “White”)				
Race: _Missing	3.13	0.906	8.278	0.038
Race: Asian	1.768	0.417	5.115	0.356
Race: Black	1.079	0.617	1.864	0.785
Race: Other	1.423	0.751	2.597	0.262
Chief complaint (CC) category (ref “administrative module”)				
CC category: diagnostic, screening, and preventive module	0.519	0.029	2.412	0.519
CC category: disease module	0.279	0.046	0.897	0.077
CC category: injuries and adverse effects module	0.454	0.11	1.234	0.184
CC category: uncodable entries	0.88	0.305	2.006	0.785
Insurance (ref “Medicaid”)				
Insurance: private	0.685	0.415	1.103	0.127
Insurance: self‐pay	0.888	0.049	4.236	0.907
Length of stay (“LOS”; continuous; hours)				
LOS	0.94	0.863	1.006	0.118
Arrival time (ref “12:01 a.m.–7:59 a.m.”)				
Arrival time: 4:01 p.m.–12:00 a.m.	0.862	0.467	1.66	0.645
Arrival time: 8:00 a.m.–4:00 p.m.	0.768	0.418	1.474	0.408
Arrival day (ref “Wednesday”)				
Arrival day: Friday	0.511	0.208	1.152	0.119
Arrival day: Monday	0.658	0.312	1.352	0.259
Arrival day: Saturday	0.771	0.357	1.606	0.493
Arrival day: Sunday	0.62	0.272	1.335	0.233
Arrival day: Thursday	0.35	0.126	0.844	0.028
Arrival day: Tuesday	0.473	0.201	1.039	0.071
Arrival month (Ref “January”)				
Arrival month: April	1.139	0.209	6.207	0.874
Arrival month: August	3.476	0.991	16.022	0.068
Arrival month: December	1.564	0.409	7.467	0.53
Arrival month: February	1.994	0.484	9.809	0.348
Arrival month: July	2.772	0.722	13.267	0.153
Arrival month: June	3.493	0.957	16.376	0.073
Arrival month: March	3.359	1.014	15.107	0.068
Arrival month: May	3.814	1.151	17.162	0.044
Arrival month: November	1.234	0.27	6.316	0.784
Arrival month: October	3.609	1.089	16.236	0.053
Arrival month: September	1.602	0.35	8.208	0.54

Multivariable logistic regression of length of stay and age association with MOID		
	OR	95% CI
Intercept	0.019	(0.008–0.039)***
Length of stay	0.936	(0.857–1.003)
Categorical age (1–5)	2.341	(1.122–5.502)*
Categorical age (6–15)	3.158	(1.492–7.489)**
Categorical age (15+)	1.447	(0.527–3.977)

*Note*: Certain analyses performed with regards to day of the week, time of the day, or month of the year were exploratory in nature to determine possible associations between system‐level factors (i.e., busyness) and MOIDs. statistically significant result for * < 0.05, ** < 0.01, *** < 0.001.

Abbreviations: MOID, missed opportunity for improving diagnosis.

## DISCUSSION

Our analysis used a set of three consensus‐based electronic triggers and revealed a MOID frequency in five pediatric EDs of 2.6% among cases at a presumed higher risk of MOIDs based on the trigger criteria. Detailed review of charts evaluated for MOIDs using the Revised Safer Dx Instrument found that approximately 50% of cases were suggestive of a MOID, of which more than half resulted in patient harm. Our analysis revealed that patients aged 1–15 years had greater odds of being classified as suggestive of a MOID when compared to patients <1 year of age. The top missed diagnoses in this cohort were brain lesions, infections, or hemorrhage; pneumonias and lung abscess; and appendicitis. Although death within 24 h of ED arrival or admission is a common quality metric,^6^, ^18^ this was not a useful trigger for identifying MOIDs in our cohort. Nevertheless, the use of electronic triggers with selective record review is an effective process to screen for harmful diagnostic errors in EDs.

Electronic triggers have been used to study diagnostic errors in various settings including primary care,^8^, ^9^, ^19^ inpatient units,^20^, ^21^ and the ED.^22^, ^23^, ^24^, ^25^, ^26^ The frequency of MOIDs identified in our study (2.6%) is half of what has been reported in prior literature,^24^, ^27^, ^28^ but similar to conclusions that MOIDs may be less frequent in higher volume pediatric EDs.^29^ Prior research has shown that return visits to the ED (Trigger 1) have a low yield in identifying cases with diagnostic error (frequency less than 3%).^30^ We built on this by adding the criteria of a return visit that results in admission. A study by Depiero et al.^31^ found a rate of missed diagnosis of 3.8% in a similar cohort of patients (returns with hospitalization) but before the use of the Safer Dx Instrument, while a study by Lam et al.^24^ reported a rate 4.8% among records identified by the electronic trigger.^24^ A study in adult patients whose return ED visits resulted in ICU admission reported high rates of diagnostic errors (13.7%)^23^ while our cohort revealed a 1.9% rate. A recent study investigating diagnostic errors in pediatric critical care found a 1.5% prevalence of diagnostic error for patients admitted to the ICU.^32^ While this study included a longer time window for the trigger criteria (up to 7 days after PICU admission), a subset of errors could be attributed to cases with diagnostic uncertainty on admission which may be a future consideration for identifying errors for patients admitted from the ED. These differences deserve investigation as they may indicate age‐specific risks. Although not specific to ED‐preventable errors, prior indicators are available that may suggest errors related to patient status (i.e., cardiorespiratory arrest), clinical assessment (i.e., change in diagnosis), or clinical management (i.e., medical emergency team activation).^21^ Incorporating indicators that are amenable to an electronic data query into our Trigger 2 may improve error identification but also may require specialized electronic refinements based on unique variables related to admission unit and clinical condition. To further reduce the burden associated with manually reviewing charts, additional refinements to the triggers focusing on symptom–disease dyads more likely to result in an error could be considered.^33^ Additionally, the ability to reliably link ED visit–based triggers with prior visits to outside sources, such as primary care,^9^ might provide improvements to trigger performance. Although prior research has reported on common rates of preventable ED patient death,^34^, ^35^ the lack of MOIDs identified via Trigger 3 in our study more closely aligns with a study by Jacobson et al.,^36^ suggesting that death may not be a strong indicator of preventable harm or error for ED patients.

Our categorization revealed that many MOIDs patients had visits relating to fever (18.4%), stomach and abdominal pain, cramps and spasms (11.8%), and labored or difficult breathing (10.5%). Many patients in our study had visits related to symptoms (85.5%), with a majority of these of symptom‐related reasons for the index visit not having the correct diagnosis in the differential list. Interestingly, on Trigger 1 return visit, we found fewer visits were categorized with the first final diagnosis related to symptoms. Further, our review noted that a differential diagnosis was documented in 75% of cases suggestive of a MOID, but the ultimate correct diagnosis was documented in the differential diagnosis list for only 30%. This highlights the role uncertainty plays in the ED diagnostic process and may support the importance of adequately considering differential diagnoses. Although it is difficult to attribute this observation to a knowledge gap or due to bias, there could be future areas for intervention especially using symptom checkers or computerized clinical decision support.

Our study found that the patient–provider interaction was the main contributing factor in over 50% of cases, which is consistent with the findings of a large global survey on diagnostic errors in the pediatric ED setting.^3^ These results were from a self‐reported survey investigating instances of diagnostic error and highlight the importance of gathering direct perspectives of those involved in error cases as reviewers in our study were often unable to ascertain a more detailed level of contributing factors based on EHR data alone. Other cases of missed diagnoses were associated with contributing factors related to the patient (21%), the system (13%), and the provider (11%). These findings suggest areas for intervention development that can improve diagnostic safety in pediatric emergency care. However, in 34% of records, the reviewers were unable to identify any contributing factors from medical record review signifying the importance of combining multiple methods to study contributory causes of diagnostic errors.

Importantly, our study found that over half of the identified cases of missed or delayed diagnoses resulted in patient harm. Of these cases, 11 (27%) required intervention, 29 (71%) required hospitalization (either initially or prolonged), and one (2%) led to permanent patient harm. A global survey of pediatric ED providers similarly reported significant levels of patient harm due to MOIDs^3^ and, though not restricted to pediatric patients^30^, ^37^ or limited to the ED setting, others have found high rates of temporary harm to patients.^20^

Our study utilized a robust methodology for chart review, employing consensus‐based triggers that were developed from our previous work. At each site, two trained pediatric emergency physicians conducted the chart reviews with an additional process for adjudication review as necessary. One strength of our study is that we did not review every chart that met the trigger criteria and potentially improves efficiency. This approach reduces the burden of a retrospective review while still providing an accurate assessment of MOIDs. Triggers play a crucial role in diagnostic safety measurement systems, as they help identify cases for further review and analysis that may be underreported or misclassified through other monitoring methods.^38^ Although voluntary reporting is an important aspect of measurement, it provides an incomplete picture of diagnostic safety. Active forms of surveillance using multiple EHR‐based triggers serves to overcome these barriers. Our study offers a method for pediatric EDs to identify diagnostic errors for quality and safety improvement. However, more research is needed to better understand whether the epidemiology of MOIDs is different in community EDs compared to EDs in children's hospitals and to provide a comparison with randomly selected medical records to determine whether electronic trigger identified MOIDs are more severe with risk of patient harm.

## LIMITATIONS

This study has several limitations. First, while our study provides a prevalence of missed opportunities based on the triggers used, we cannot estimate the overall prevalence of diagnostic errors in pediatric EDs. Second, the participating sites are limited to pediatric academic settings so the reported errors may not apply to other settings such as community EDs. However, most institutions have return visits and deaths following an ED visit as a quality indicator, which provides face validity and the potential for wider adaptation across general and pediatric EDs. Third, as a retrospective study, the reviewers were limited to the information available in the EHR, which may have gaps in the original documentation by providers or introduce hindsight bias inherent in this study design. Although we had planned on collecting more detailed subcategories of possible MOID contributory factors, reviewers were unable to consistently collect information retrospectively from the EHR to provide insight beyond major factor category (patient, provider, system, patient–provider). To collect this information, refinement to subcategory options should consider items most likely to be available in the EHR or most applicable to possible intervention. Key informant interviews with providers who cared for the patients could likely contribute to a more complete understanding of how diagnostic errors occur. Additionally, due to the chart screening process, charts with missed opportunities may have been excluded, resulting in an underreporting of the number of missed opportunities in this population. However, our approach of identifying a variety of reasons to exclude a chart from further review could also translate into natural language processing–based queries to further reduce the burden of manual review in instances where a MOID is very unlikely (i.e., unrelated visits or progression of the same condition). There is also a possibility of reviewer bias in interpreting whether a MOID was present. To mitigate this, we used comprehensive procedures to train reviewers, held regular study meetings with reviewers to discuss challenges between sites, and ensured consistent chart reviews using the Revised Safer Dx Instrument. Further, an adjudication process was used when it was unclear if MOIDs occurred (i.e., a Safer Dx score of 4). However, we noted a wide variation range in MOIDs (0.6%–4.6%) and “unlikely for MOIDS” (91%–98%) across sites. While we mitigated reviewer bias by thorough reviewer training and ensured consistency using the Revised Safer Dx Instrument, reluctance may exist even among experienced reviewers to assign an error on a chart review. Fourth, we did not explore other planned methods for identifying missed opportunities, such as cases referred for review or patient complaints, as we found them unsuitable for electronic trigger implementation. Considering the low prevalence of missed opportunities identified through these triggers alone, trigger tools should be considered as only one method in a broader context of tools used to identify diagnostic errors. Fifth, no controls for Type 1 error rate were performed for the simple and multivariable logistic regression models. The low rate of MOID frequency for the entire cohort and within triggers limited statistical power and the ability to adjust the Type 1 error rate with corrections such as the Bonferroni correction. This can increase the likelihood of spurious associations and results should be interpreted within this context. Sixth, this study primarily examined simple correlations, which are vulnerable to confounding and may be inflated or spurious, especially in a nonrandom sample. We encourage future studies to consider holistic models.

## CONCLUSIONS

Our analysis of a large, multicenter cohort of pediatric emergency visits revealed deeper insights into the frequency, type, causative factors, and severity of diagnostic errors. Recognizing that use of triggers will identify a narrow spectrum of diagnostic errors, our methods can be refined to improve their performance. Furthermore, after refinement, triggers can be used as a patient safety toolkit to monitor for diagnostic quality.

## AUTHOR CONTRIBUTIONS

Prashant Mahajan and Hardeep Singh contributed to the study concept and design. Prashant Mahajan, Kathy Shaw, James Chamberlain, Richard M. Ruddy, and Elizabeth R. Alpern oversaw acquisition of the data. Prashant Mahajan, Sarah J. Parker, Gregor Horvath, and Giselle Kolenic performed analysis and interpretation of the data. Prashant Mahajan and Sarah J. Parker drafted the manuscript. All authors contributed critical revisions of the manuscript for important intellectual content. Emily White, Elizabeth Freiheit, Gregor Horvath, and Giselle Kolenic provided statistical expertise. Prashant Mahajan and Hardeep Singh acquired funding for the project.

## FUNDING INFORMATION

Dr. Singh is supported in part by the Houston Veterans Administration (VA) Health Systems Research and Development (HSR&D) Center for Innovations in Quality, Effectiveness and Safety (CIN13–413), the VA National Center for Patient Safety, and the Agency for Healthcare Research and Quality (R01HS028595, R18HS029347, and R01HS029318). This project content is solely the responsibility of the authors and does not necessarily represent the official views of the Agency for Healthcare Research and Quality.

## CONFLICT OF INTEREST STATEMENT

The authors declare no conflicts of interest.

## Supporting information


Data S1.

Data S2.

Data S3.

Data S4.

Data S5.

Data S6.

## Data Availability

The data that supports the findings of this study are available in the supplementary material of this article.
