# Supplementary material for: Epidemiology of diagnostic errors in pediatric emergency departments using electronic triggers
Source: Acad Emerg Med. 2025 Jan 15;32(3):226–45. doi: 10.1111/acem.15087 (PMC11921087; doi:10.1111/acem.15087)
Supplement: Supplementary file 1 — Data S1. [file ACEM-32-226-s006.docx]

**Supplementary material 1.** Participating site details for patients ≤ 21 years old (year 2019)

| Site | Emergency Department annual visits | Hospital admission | Intensive care unit admission |
| --- | --- | --- | --- |
| 1 | 35,000 | 17% | 3% |
| 2 | 57,000 | 18% | 2% |
| 3 | 97,000 | 18% | 1% |
| 4 | 101,000 | 19% | 1% |
| 5 | 98,000 | 21% | 1% |
