# Supplementary material for: Epidemiology of diagnostic errors in pediatric emergency departments using electronic triggers
Source: Acad Emerg Med. 2025 Jan 15;32(3):226–45. doi: 10.1111/acem.15087 (PMC11921087; doi:10.1111/acem.15087)
Supplement: Supplementary file 2 — Data S2. [file ACEM-32-226-s002.docx]

**Supplementary material 2.** Data query

/******************************************************************************************************************************************************************

/*

(c) 2019 The Regents of the University of Michigan.

All rights reserved. Further use or dissemination is strictly prohibited without the express written consent of the University of Michigan.

MOIDS- Trigger 1 -

Unscheduled Return Visits within 10 Days Resulting in Admission

MOIDS- Trigger 2

Care Escalation to ICU within 24 hours

MOIDS- Trigger 3 -

All Deaths in the ED and within 24 Hours of ED Departure Time

DETAILS: The base cohort code is from PECARN Registry data submission that is used across PECARN Registry sties.

Notes have been made where site modifications will be required.

Customization required:

1. Will need to define the ED Department

2. Determine the name of ED_events in the ED Event Records

3. Need to deterimine how/if to exclude test patients if relevant at your site.

4. Need to determine hospital classes and admission_types to include for return visits (inpatient, observation)

Created: 5.16.2019

Updated: 8.24.2020 by Chiu-Mei Chen

******************************************************************************************************************************************************************/

--truncate table moids_cohort;

--insert into moids_cohort

DROP TABLE moids_cohort;

CREATE TABLE moids_cohort AS

WITH

hsp_qry AS

(SELECT /*materialize*/

hsp.pat_id,

pat.pat_mrn_id ,

pat.birth_date,

EPIC_UTIL.EFN_DATEDIFF('ageyears',pat.birth_date,hsp.adt_arrival_time ) age_year ,

sex.name AS sex,

ZC_PATIENT_RACE.TITLE AS RACE,

ETHNIC.TITLE AS ETHNICITY,

FC.FINANCIAL_CLASS_NAME,

hsp.pat_enc_csn_id ,

hsp.hsp_account_id,

acct.adm_date_time,

acct.acct_class_ha_c,

--zacct.name AS account_class,

pt_c.name AS hosp_admission_status,

admtype.name AS admit_type,

hsp.HOSP_ADMSN_TIME,

hsp.inp_adm_date,

hsp.adt_arrival_time,

hsp.ed_departure_time as ED_DEPARTURE_DTTM,--same as ed datamart ED_DEPARTURE_DTTM;same as ed_iev_event_info.event_time where event_type in ('60', '1600090312', '65')

hsp.hosp_disch_time,

nvl(case when hsp.hosp_disch_time>hsp.ed_departure_time OR hsp.ed_departure_time IS NULL then ROUND((hsp.hosp_disch_time-NVL(hsp.ed_departure_time,hsp.HOSP_ADMSN_TIME))*24,1) end,0) AS HOSP_LOS_HRS,

hsp.ed_episode_id,

eddisp.name AS ED_DISPOSITION_NAME ,

hspdisp.name hsp_disposition,

hsp.acuity_level_c,

hsp.disch_disp_c,

case

when hsp.ed_episode_id IS NOT NULL then 'ED' --this rule is applied to t1 index and t2 and t3

when HSD_BASE_CLASS_MAP.acct_class_map_c=104 and --Observation

hsp.ADT_PATIENT_STAT_C in (2,3) then 'IP'--2 Admission;3 Discharged

when hsp.ADT_PATIENT_STAT_C=6 then 'OP'--Hospital Outpatient Visit

else zc_acct_basecls_ha.abbr END AS pat_class,

NVL(pat2.DEATH_TM,pat.DEATH_DATE) as death_date

FROM pat_enc_hsp hsp

INNER JOIN patient pat ON hsp.pat_id = pat.pat_id

inner JOIN PATIENT_2 pat2 ON pat.PAT_ID=pat2.PAT_ID

inner join pat_enc_2 enc2 on hsp.pat_enc_csn_id=enc2.pat_enc_csn_id

--INNER JOIN zc_disch_disp stat ON hsp.disch_disp_c = stat.disch_disp_c

INNER JOIN hsp_account acct ON hsp.hsp_account_id = acct.hsp_account_id

--LEFT JOIN zc_acct_class_ha zacct ON acct.acct_class_ha_c = zacct.acct_class_ha_c

LEFT JOIN ZC_DISCH_DISP hspdisp ON hsp.disch_disp_c = hspdisp.disch_disp_c

LEFT JOIN zc_ed_disposition eddisp ON hsp.ed_disposition_c = eddisp.ed_disposition_c

LEFT JOIN zc_sex sex ON pat.sex_c = sex.rcpt_mem_sex_c

-------RACE

LEFT JOIN

PATIENT_RACE PR

ON PR.PAT_ID = PAT.PAT_ID

AND PR.LINE= 1

LEFT JOIN

ZC_PATIENT_RACE

ON ZC_PATIENT_RACE.PATIENT_RACE_C= PR.PATIENT_RACE_C

---------ETHNIC-----------

LEFT JOIN

ZC_ETHNIC_GROUP ETHNIC

ON ETHNIC.ETHNIC_GROUP_C =PAT.ETHNIC_GROUP_C

--------INSURANCE--------

LEFT JOIN CLARITY.HSP_ACCT_CVG_LIST CVGL --PRIMARY INSURANCE

ON CVGL.HSP_ACCOUNT_ID=HSP.HSP_ACCOUNT_ID

AND CVGL.LINE=1

LEFT JOIN CLARITY.COVERAGE CVG

ON CVG.COVERAGE_ID= CVGL.COVERAGE_ID

LEFT JOIN CLARITY_EPP BENPLAN ON

CVG.PLAN_ID = BENPLAN.BENEFIT_PLAN_ID

LEFT JOIN CLARITY_FC FC

ON FC.FINANCIAL_CLASS=BENPLAN.CLM_FIN_CL_C

LEFT JOIN ZC_MC_ADM_TYPE ADMTYPE ON ADMTYPE.ADMISSION_TYPE_C = ACCT.ADMISSION_TYPE_C

LEFT OUTER JOIN zc_pat_class pt_c ON hsp.adt_pat_class_c = pt_c.adt_pat_class_c

LEFT JOIN HSD_BASE_CLASS_MAP ON --FOR PAT_CLASS

ENC2.ADT_PAT_CLASS_C=HSD_BASE_CLASS_MAP.ACCT_CLASS_MAP_C

AND ((HSD_BASE_CLASS_MAP.PROFILE_ID IS NULL ) OR (HSD_BASE_CLASS_MAP.PROFILE_ID=1))

LEFT JOIN clarity.ZC_ACCT_BASECLS_HA ZC_ACCT_BASECLS_HA ON

HSD_BASE_CLASS_MAP.BASE_CLASS_MAP_C=ZC_ACCT_BASECLS_HA.ACCT_BASECLS_HA_C

WHERE

COALESCE(hsp.adt_arrival_time,hsp.HOSP_ADMSN_TIME,hsp.hosp_disch_time,hsp.exp_admission_time) between

TO_DATE ('20180101 00:00:00', 'YYYYMMDD HH24:MI:SS') AND

TO_DATE ('20191231 23:59:59', 'YYYYMMDD HH24:MI:SS')+10

AND nvl(hsp.admit_conf_stat_c,0) <>3 --Excluding only Cancelled records

AND (eddisp.name is null or

( eddisp.name NOT IN

( 'ED Dismiss - Never Arrived',

'Expected - Never Arrived',

'Left Without Being Seen' ,

'Send to Psych Emergency',

'Send to L'||'&'||'D',

'Send to Adult Emergency',

'ED Protocol',

'ACE',

'PES ONLY - Discharge to Partial Hospital',

'Pending'

)

AND eddisp.name NOT LIKE 'Nurse Only%'

AND eddisp.name NOT LIKE 'Transfer to%'

AND eddisp.name NOT LIKE 'Triage to%'

)

)

AND NOT EXISTS

(SELECT 1 --eliminate test patients

FROM umhs_custom.um_test_patient_mv tp

WHERE pat.pat_id = tp.pat_id) -- Exclude Test patients

),

--Clarity ADT to get the first Admission Department--

adt as (

select

hsp.pat_enc_csn_id,

dep.department_name,

ROW_NUMBER () OVER (PARTITION BY hsp.pat_enc_csn_id ORDER BY adt.effective_time asc) AS max_row--get the first dept

from hsp_qry hsp

inner join clarity_adt adt on adt.pat_enc_csn_id = hsp.pat_enc_csn_id

inner join clarity_dep dep on dep.department_id = adt.department_id

where

adt.event_type_c =1 and --admission

adt.EVENT_SUBTYPE_C <> 2--cancel

),

ED_last_arvl_data as --time is Corrected by coding & abstracting.Some adt_arrival_time is not correct

(

SELECT

mrs.pat_enc_csn_id,

ed_event_history.event_audit_time,

ROW_NUMBER () OVER (PARTITION BY mrs.pat_enc_csn_id ORDER BY e.event_time DESC, e.event_id DESC, e.line DESC, ed_event_history.event_audit_time, ed_event_history.line) AS min_audit_row

FROM hsp_qry mrs

INNER JOIN ed_iev_pat_info ep ON mrs.pat_enc_csn_id = ep.pat_csn

INNER JOIN ed_iev_event_info e ON ep.event_id = e.event_id

INNER JOIN clarity_dep dept ON e.event_dept_id = dept.department_id

INNER JOIN ed_event_history ON e.event_id = ed_event_history.event_id

AND ed_event_history.event_type_audit = 50--arrival

WHERE

MRS.pat_class='ED' and

event_cmt = 'Corrected by coding ' || CHR (38) || ' abstracting.' and

e.event_type =50 --Patient arrived in ED

AND e.event_id IS NOT NULL AND e.event_status_c IS NULL AND

e.event_dept_id IN (100011101, 100011801, 100051801) --PES-UH,AES-UH, CES-MH

) ,

reason_for_visit as

(

select

enc.pat_enc_csn_id,

LISTAGG(v.REASON_VISIT_NAME , ';') WITHIN GROUP (ORDER BY line) as CComplaints,

LISTAGG (rsn.comments, '; ') WITHIN GROUP (ORDER BY line) as ComplaintComments

from hsp_qry enc

inner join

PAT_ENC_RSN_VISIT rsn --Deprecated Columns in 2018 upgrade

on enc.PAT_ENC_CSN_ID=rsn.PAT_ENC_CSN_ID

inner join CL_RSN_FOR_VISIT v

on v.reason_visit_id=rsn.enc_reason_id

WHERE PAT_CLASS IN ('ED','IP')-- This sub query is for Index_CComplaints and Return_CComplaints so we have to apply both 'ED' and 'IP'.

group by enc.pat_enc_csn_id

),

-------below is for Return_Unit: Inpatient Unit of Return Hospital Admission

------get first admit department except ED

Transfer_to as

(

select

HSP.PAT_ENC_CSN_ID,

min(adt.effective_time) as effective_time

from hsp_qry hsp

inner join clarity_adt adt on adt.pat_enc_csn_id = hsp.pat_enc_csn_id

inner join clarity_dep dep on dep.department_id = adt.department_id

-- Get Min transfers to any unit OTHER than the ED

where

adt.event_type_c in (1,3) and EVENT_SUBTYPE_C <> 2--EVENT_TYPE: admission and transfer in ;SUBYPE_C 2 - Canceled

AND

DEP.department_id NOT IN --Some patients were transferred from adult ED to Peds ED , or vice versa, and we don’t want to get ED as first admit department so we exclude all ED departments and temporary lounge units here.

(100011801, --AES-UH

100051801, --CES-MH

100011101, --PES-UH

910199001, --UADM Admitting lounge UH

910199030) --MH ABCC Admitting lounge MH

-- Event is an Admission or Transfer and not cancelled

group by HSP.PAT_ENC_CSN_ID

),

to_next_unit as

(

select

distinct

tounit.PAT_ENC_CSN_ID,

tounit.effective_time as NEXT_UNIT_DTTM,

dep.DEPT_ABBREVIATION AS NEXT_UNIT

from transfer_to tounit

inner join clarity_adt adt on adt.effective_time = tounit.effective_time and

adt.pat_enc_csn_id = tounit.pat_enc_csn_id

inner join clarity_dep dep on adt.department_id = dep.department_id

where

adt.event_type_c in (1,3) and EVENT_SUBTYPE_C <> 2--EVENT_TYPE: admission and transfer in ;SUBYPE_C 2 - Canceled

and adt.department_id NOT IN

(100011801, --AES-UH

100051801, --CES-MH

100011101, --PES-UH

910199001, --UADM Admitting lounge UH

910199030)

),

-------------------------

----------below is for dx

-------------------------

ed_dx_qry AS

(SELECT

DISTINCT dx.pat_enc_csn_id pat_enc_csn_id,

dx.dx_id,

icd10.line AS icdline,

dx.DX_ED_YN,

dx.primary_dx_yn,

edg.dx_name,

icd10.code icd_code,

RANK()OVER(PARTITION BY dx.pat_enc_csn_id ORDER BY dx.pat_enc_csn_id,case when PRIMARY_DX_YN='Y' then 1 else 0 end desc,dx.line asc NULLS LAST) as row_NUM --pick primary first and tehn first line #

FROM hsp_qry hsp

INNER JOIN pat_enc_dx dx ON hsp.pat_enc_csn_id = dx.pat_enc_csn_id -- restricts to primary ED Clinical Impression;--1 is not necesary primary

INNER JOIN clarity_edg edg ON dx.dx_id = edg.dx_id

INNER JOIN edg_current_icd10 icd10 ON dx.dx_id = icd10.dx_id AND icd10.line = 1 -- Some dx_name are coded with two distinct ICD_10 codes, this picks up the first ICD10 code coded to a particular dx_name

WHERE dx.dx_ed_yn = 'Y'),

-- get primary ED dx (clinical impression) for each visit. ED Clinical Impressions are determined by the flag dx_ed_yn. A patient may not have any ED Clinical Impressions flagged on the encounter.

-- Additionally, not all ED Clinical impressions have a primary. For the purposes of this pull, we have retricted on dx_ed_yn to only pull the ED Clinical Impressions.

-- Some patients will have more than one ED clinical imporession, and in these cases the first clinical impression is pulled.

--get hosptial admission diagnosis (where applicable)

hspadm_dx_qry AS

(SELECT DISTINCT CAST (dx.pat_enc_csn_id AS INT) pat_enc_csn_id,

ROW_NUMBER () OVER (PARTITION BY dx.pat_enc_csn_id ORDER BY dx.line ) AS row_num,

dx.dx_id,

icd10.line AS icdline,

edg.dx_name,

icd10.code icd_code,

dx.ADMIT_DIAG_TEXT

FROM hsp_qry hsp

INNER JOIN hsp_admit_diag dx

ON hsp.pat_enc_csn_id = dx.pat_enc_csn_id

--AND dx.line = '1' -- restricts to primary/first on record; some of the first one is line=2, no line=1

inner JOIN clarity_edg edg ON dx.dx_id = edg.dx_id

inner JOIN edg_current_icd10 icd10 ON dx.dx_id = icd10.dx_id AND

icd10.line = '1'), -- This picks up the first ICD10 code, coded on record

--get final diagnosis (primary billing diagnosis) for each visit

final_dx_qry AS

(SELECT CAST (hsp.pat_enc_csn_id AS INT) pat_enc_csn_id,

CAST (dx.line AS INT) line,

ROW_NUMBER () OVER (PARTITION BY hsp.pat_enc_csn_id ORDER BY dx.line ) AS row_num,

edg.ref_bill_code icd_code,

edg.dx_name,

edg.dx_group,

edg.ref_bill_code_set_c

FROM hsp_qry hsp

INNER JOIN hsp_acct_dx_list dx ON hsp.hsp_account_id = dx.hsp_account_id --AND dx.line = 1

INNER JOIN clarity_edg edg ON dx.dx_id = edg.dx_id

WHERE REF_BILL_CODE_SET_C = 2),

final_dx as

(

select

pat_enc_csn_id,

LISTAGG(dx_name, ';') WITHIN GROUP (ORDER BY row_NUM) as final_DX_LIST,

LISTAGG(icd_code, ';') WITHIN GROUP (ORDER BY row_NUM) as final_ICD10_LIST

from final_dx_qry

group by

pat_enc_csn_id

),

----------------------

------below is for icu

----------------------

stg_admit_info as

(SELECT

MRS.pat_enc_csn_id,

adt_in.xfer_event_id,

adt_in.effective_time,

adt_in.event_id,

adt_in.next_out_event_id,

dep.dept_abbreviation icu_dept,

dep.department_id,

dep.specialty,

dept2.icu_dept_yn,

ROW_NUMBER () OVER (PARTITION BY MRS.pat_enc_csn_id ORDER BY adt_in.effective_time, adt_in.event_id) AS min_time

FROM hsp_qry mrs

INNER JOIN clarity_adt adt_in ON mrs.pat_enc_csn_id = adt_in.pat_enc_csn_id

INNER JOIN clarity_dep dep ON adt_in.department_id = dep.department_id

INNER JOIN clarity_dep_2 dept2 ON dept2.department_id = dep.department_id

WHERE

(MRS.pat_class='ED' )

AND adt_in.event_type_c IN (1,3) --admission and transfer in

AND adt_in.event_subtype_c IN (1,3) --original, update

AND adt_in.department_id NOT IN (100011801, --AES-UH

100051801, --CES-MH

100011101, --PES-UH

910199001, --UADM Admitting lounge UH

910199030)

), --MH ABCC Admitting lounge MH

icu as

(

SELECT --UEDC_2529401 find admission to ICU times

DISTINCT

admit_info.pat_enc_csn_id,

FIRST_VALUE(admit_info.effective_time) IGNORE NULLS OVER (PARTITION BY admit_info.pat_enc_csn_id ORDER BY min_time ROWS BETWEEN UNBOUNDED PRECEDING AND UNBOUNDED FOLLOWING) first_ICU_dttm,

FIRST_VALUE(admit_info.icu_dept) IGNORE NULLS OVER (PARTITION BY ADMIT_INFO.pat_enc_csn_id ORDER BY min_time ROWS BETWEEN UNBOUNDED PRECEDING AND UNBOUNDED FOLLOWING) first_icu_unit_name

FROM stg_admit_info admit_info

WHERE

admit_info.icu_dept_yn = 'Y' --ICU rooms

)

select

hsp_qry.*,

nvl(eed_arrival.event_audit_time,hsp_qry.adt_arrival_time) AS ED_ARRIVAL_DTTM ,

--eed_not_arrival.ed_discharge_time ,

icu.FIRST_ICU_DTTM ,

icu.FIRST_ICU_UNIT_NAME ,

reason_for_visit.CComplaints,

reason_for_visit.complaintComments,

n.next_unit,

n.NEXT_UNIT_DTTM ,

eddx.dx_name as ED_ClinicalImpression ,

eddx.icd_code as ED_ClinicalImpression_ICD10 ,

adx.dx_name as Hosp_Admit_Dx,

adx.icd_code as Hosp_Admit_ICD10 ,

fdx.dx_name as Final_Dx ,

fdx.icd_code as Final_ICD10,

fd.final_DX_LIST,

fd.final_ICD10_LIST,

ADT.department_name AS admit_dept

from hsp_qry

left join

reason_for_visit

on hsp_qry.pat_enc_csn_id=reason_for_visit.pat_enc_csn_id

LEFT JOIN ED_last_arvl_data eed_arrival ON hsp_qry.pat_enc_csn_id = eed_arrival.pat_enc_csn_id

and min_audit_row = 1 --The ED arrival time may be corrected a couple times, so we pick the last event time.

left join icu on icu.pat_enc_csn_id=hsp_qry.pat_enc_csn_id

left join to_next_unit n on n.pat_enc_csn_id=hsp_qry.pat_enc_csn_id

LEFT JOIN ed_dx_qry eddx ON eddx.pat_enc_csn_id = hsp_qry.pat_enc_csn_id and eddx.row_num=1

LEFT JOIN hspadm_dx_qry adx ON adx.pat_enc_csn_id = hsp_qry.pat_enc_csn_id and adx.row_num=1

LEFT JOIN final_dx_qry fdx ON fdx.pat_enc_csn_id = hsp_qry.pat_enc_csn_id and fdx.row_num=1

LEFT JOIN final_dx fd ON fd.pat_enc_csn_id = hsp_qry.pat_enc_csn_id

LEFT JOIN adt on adt.pat_enc_csn_id = hsp_qry.pat_enc_csn_id and adt.max_row=1

where

hsp_qry.pat_class in ('ED','IP') ;

commit;

----------------------------------------

----------Trigger1----------------------------

----------------------------------------

drop table moids_t1;

create table moids_t1 as

SELECT

DISTINCT

ed.pat_mrn_id MRN,

ed.age_YEAR age,

ed.sex,

ed.RACE,

ed.ETHNICITY,

ed.FINANCIAL_CLASS_NAME,

ed.pat_enc_csn_id AS Index_CSN,

rv.pat_enc_csn_id AS RV_CSN,

nvl(ed.ED_ARRIVAL_DTTM,ed.adt_arrival_time) AS Index_DT,

ed.ED_DEPARTURE_DTTM AS Index_Depart_DT,

NVL(rv.ED_ARRIVAL_DTTM,rv.HOSP_ADMSN_TIME) AS Return_DT,

round((NVL(rv.ED_ARRIVAL_DTTM,rv.HOSP_ADMSN_TIME) - ed.ED_DEPARTURE_DTTM) * 24,1) AS return_hours,

rv.next_unit as Return_Unit,

ed.ed_disposition_NAME AS Index_EDDisposition,

rv.ed_disposition_NAME AS Return_EDDisposition,

rv.admit_dept as Return_Type,

ed.CComplaints AS Index_CComplaints,

ed.ComplaintComments AS Index_ComplaintComments,

ed.ED_ClinicalImpression AS Index_ClinicalImpression,

ed.ED_ClinicalImpression_ICD10 as Index_ClinicalImpression_ICD10,

ed.Hosp_Admit_Dx AS Index_HospAdmit_Dx,

ed.Hosp_Admit_ICD10 AS Index_HospAdmit_ICD10,

ed.Final_Dx AS Index_FinalDx,

ed.Final_ICD10 AS Index_Final_ICD10,

ED.Final_Dx_LIST AS Index_FinalDx_LIST,

ED.Final_ICD10_LIST AS Index_Final_ICD10_LIST,

rv.CComplaints AS Return_CComplaints,

rv.ComplaintComments AS Return_ComplaintComments,

rv.ED_ClinicalImpression AS Return_ClinicalImpression,

rv.ED_ClinicalImpression_ICD10 AS Return_ClinicalImpression_ICD10,

rv.Hosp_Admit_Dx AS Return_HospAdmit_DX,

rv.Hosp_Admit_ICD10 AS Return_HospAdmit_ICD10,

rv.Final_DX AS Return_FinalDx,

rv.Final_ICD10 AS Return_Final_ICD10,

RV.Final_Dx_LIST AS Return_FinalDx_LIST,

RV.Final_ICD10_LIST AS Return_Final_ICD10_LIST,

ROUND((ED.ED_DEPARTURE_DTTM-nvl(ed.ED_ARRIVAL_DTTM,ed.adt_arrival_time))*24,1) AS INDEX_ED_LOS_HRS,

ROUND(NVL(ED.HOSP_LOS_HRS,0),1) as INDEX_HOSP_LOS_HRS,

ROUND((RV.ED_DEPARTURE_DTTM-nvl(RV.ED_ARRIVAL_DTTM,RV.adt_arrival_time))*24,1) AS RV_ED_LOS_HRS,

ROUND(NVL(RV.HOSP_LOS_HRS,0),1) AS RV_HOSP_LOS_HRS

--RANK()OVER(PARTITION BY ed.pat_enc_csn_id ORDER BY ed.pat_enc_csn_id,NVL(rv.ED_ARRIVAL_DTTM,rv.HOSP_ADMSN_TIME) asc) as row_NUM --pick first return

FROM moids_cohort ed

INNER JOIN moids_cohort rv

ON ed.pat_id = rv.pat_id

where

ed.pat_class='ED' and

(rv.pat_class='IP' or

rv.ed_disposition_name='Admit' or

RV.hosp_admission_status in

(

'Inpatient',

'Obs Greater than 48 Hours'--,'Observation'

) OR

(RV.hosp_admission_status NOT IN ('Outpatient','Hospital Outpatient Surgery') AND RV.HOSP_LOS_HRS>0)

)

AND (NVL(rv.ED_ARRIVAL_DTTM,rv.HOSP_ADMSN_TIME) > ed.ED_DEPARTURE_DTTM)

AND ((NVL(rv.ED_ARRIVAL_DTTM,rv.HOSP_ADMSN_TIME) - ed.ED_DEPARTURE_DTTM) * 24) < 240 AND

(

(NVL(rv.ED_ARRIVAL_DTTM,rv.HOSP_ADMSN_TIME) - ed.ED_DEPARTURE_DTTM) * 24 >=4 OR--Some patients were admitted from ED but this admission was associated with a new CSN , not original ED CSN, so I set up this 4-hours rule to not count this admission as “return” admission. If next admission is within 4 hours and ED disposition is not “discharge” then it’s very possible that this admission is actually connected to the ED visit, not return.

ed.ed_disposition_NAME like '%Discharge%'-- If index ed_disposition_NAME is discharge then we can tell the next admission is return visit.

)

AND rv.admit_type in ('Urgent','Emergency')

and to_char(nvl(ed.ED_ARRIVAL_DTTM,ed.adt_arrival_time),'yyyy') in ('2018','2019');

commit;

----------------

--------T2

---MOIDS- Trigger 2

---Care Escalation to ICU within 24 hours-----

----------------------

DROP TABLE MOIDS_T2;

CREATE TABLE MOIDS_T2 AS

select distinct

hsp.pat_id,

hsp.pat_mrn_id as MRN,

hsp.age_year as age,

hsp.sex,

hsp.RACE,

hsp.ETHNICITY,

hsp.FINANCIAL_CLASS_NAME,

hsp.pat_enc_csn_id as CSN,

nvl(hsp.ED_ARRIVAL_DTTM,hsp.adt_arrival_time) Index_DT,

hsp.ED_DEPARTURE_DTTM Index_Discharge_DT,

hsp.Next_Unit,

FIRST_ICU_UNIT_NAME ICU_Unit,

round((FIRST_ICU_DTTM-ED_DEPARTURE_DTTM)*24,1) as Time_to_ICU_Hours,

hsp.ed_disposition_NAME AS Index_EDDisposition,

hsp_disposition as Index_HospDisposition,

CComplaints AS Index_CComplaints,

ComplaintComments AS Index_ComplaintComments,

ED_ClinicalImpression AS Index_ED_ClinicalImpression,

ED_ClinicalImpression_ICD10 as Index_ED_ClinicalImpression_ICD10,

Hosp_Admit_Dx AS Index_HospAdmit_Dx,

Hosp_Admit_ICD10 AS Index_HospAdmit_ICD10,

Final_Dx AS Index_FinalDx,

Final_ICD10 AS Index_Final_ICD10,

HSP.Final_Dx_LIST AS Index_FinalDx_LIST,

HSP.Final_ICD10_LIST AS Index_Final_ICD10_LIST,

ROUND((HSP.ED_DEPARTURE_DTTM-nvl(HSP.ED_ARRIVAL_DTTM,HSP.adt_arrival_time))*24,1) AS INDEX_ED_LOS_HRS,

NVL(ROUND(HSP.HOSP_LOS_HRS,1),0) as INDEX_HOSP_LOS_HRS

from MOIDS_COHORT hsp

WHERE

PAT_CLASS='ED' AND

(FIRST_ICU_DTTM-ED_DEPARTURE_DTTM)*24<=24 AND

NEXT_UNIT<>FIRST_ICU_UNIT_NAME and

NEXT_UNIT NOT LIKE 'OPERRM%' AND--Eliminate ED TO OR TO ICU path

to_char(nvl(hsp.ED_ARRIVAL_DTTM,hsp.adt_arrival_time),'yyyy') in ('2018','2019');

---------------------------

-------------All Deaths in the ED and within 24 Hours of ED Departure Time

-------------------T3

DROP TABLE MOIDS_T3;

CREATE TABLE MOIDS_T3 AS

select distinct

hsp.pat_mrn_id as MRN,

hsp.age_year as age,

hsp.sex,

hsp.RACE,

hsp.ETHNICITY,

hsp.FINANCIAL_CLASS_NAME,

hsp.pat_enc_csn_id as CSN,

nvl(hsp.ED_ARRIVAL_DTTM,hsp.adt_arrival_time) Index_DT,

DEATH_DATE,

hsp.ED_DEPARTURE_DTTM Index_Depart_DT,

HSP.hosp_disch_time Index_Hosp_Discharge_DT,

(ED_DEPARTURE_DTTM-DEATH_DATE)*24 Discharge_to_Death,

ed_disposition_NAME AS Index_EDDisposition,

hsp_disposition as Index_HospDisposition,

CComplaints AS Index_CComplaints,

ComplaintComments AS Index_ComplaintComments,

ED_ClinicalImpression AS Index_ED_ClinicalImpression,

ED_ClinicalImpression_ICD10 as Index_ED_ClinicalImpression_ICD10,

Hosp_Admit_Dx AS Index_HospAdmit_Dx,

Hosp_Admit_ICD10 AS Index_HospAdmit_ICD10,

Final_Dx AS Index_FinalDx,

Final_ICD10 AS Index_Final_ICD10,

HSP.Final_Dx_LIST AS Index_FinalDx_LIST,

HSP.Final_ICD10_LIST AS Index_Final_ICD10_LIST,

ROUND((HSP.ED_DEPARTURE_DTTM-nvl(HSP.ED_ARRIVAL_DTTM,HSP.adt_arrival_time))*24,1) AS INDEX_ED_LOS_HRS,

NVL(ROUND(HSP.HOSP_LOS_HRS,1),0) as INDEX_HOSP_LOS_HRS

from MOIDS_COHORT hsp

WHERE

PAT_CLASS='ED' AND

(

ED_DISPOSITION_NAME ='Deceased' OR

(DEATH_DATE-ED_DEPARTURE_DTTM)*24<=24

) AND

TRUNC(DEATH_DATE)>=TRUNC(nvl(hsp.ED_ARRIVAL_DTTM,hsp.adt_arrival_time)) AND--SOME DEATH DATES ARE NOT CORRECT SO ADDED THIS RULE

to_char(nvl(hsp.ED_ARRIVAL_DTTM,hsp.adt_arrival_time),'yyyy') in ('2018','2019');
