# Supplementary material for: Epidemiology of diagnostic errors in pediatric emergency departments using electronic triggers
Source: Acad Emerg Med. 2025 Jan 15;32(3):226–45. doi: 10.1111/acem.15087 (PMC11921087; doi:10.1111/acem.15087)
Supplement: Supplementary file 3 — Data S3. [file ACEM-32-226-s001.docx]

**Supplementary material 3.** Data Flow Chart
