# Supplementary figures and images for: Epidemiology of diagnostic errors in pediatric emergency departments using electronic triggers
Source: Acad Emerg Med. 2025 Jan 15;32(3):226–45. doi: 10.1111/acem.15087 (PMC11921087; doi:10.1111/acem.15087)

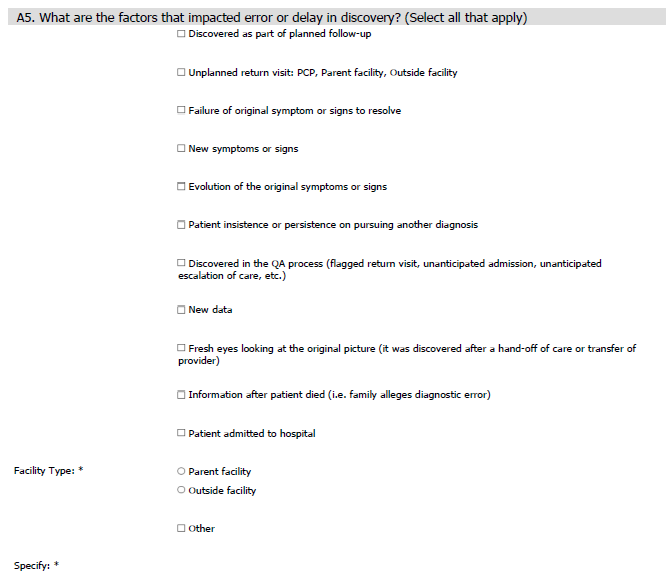


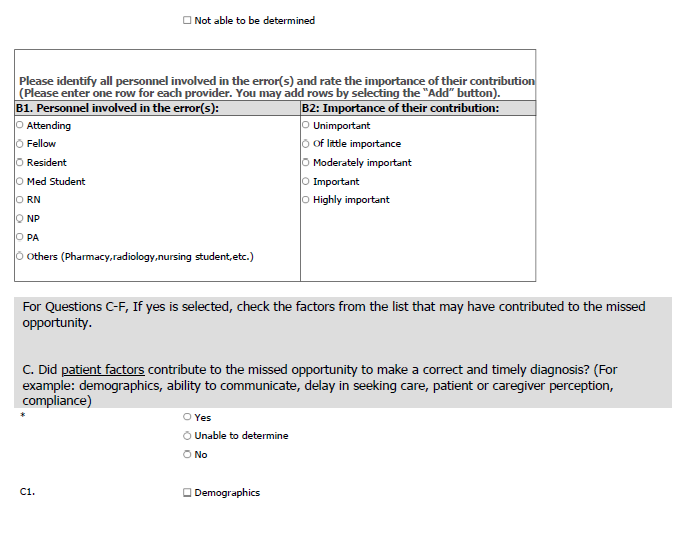


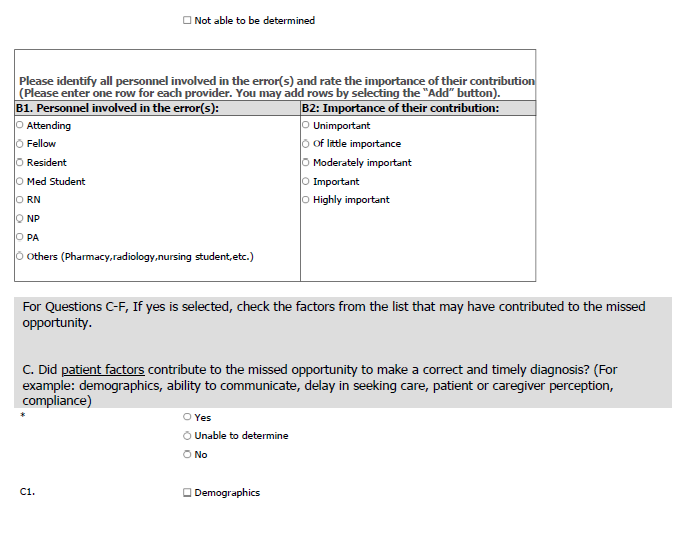


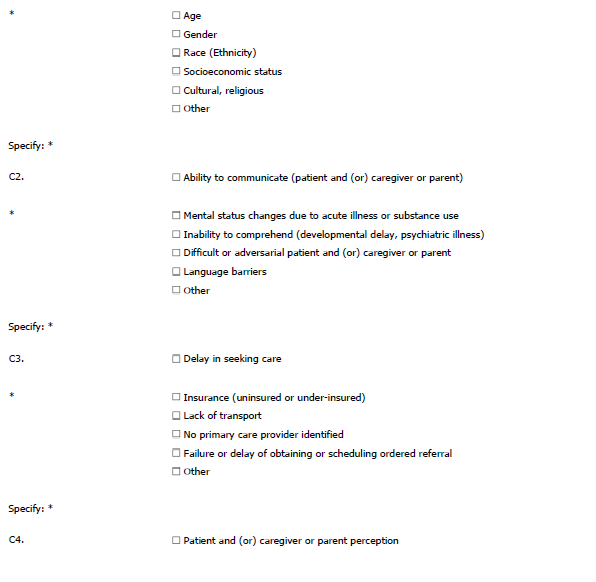


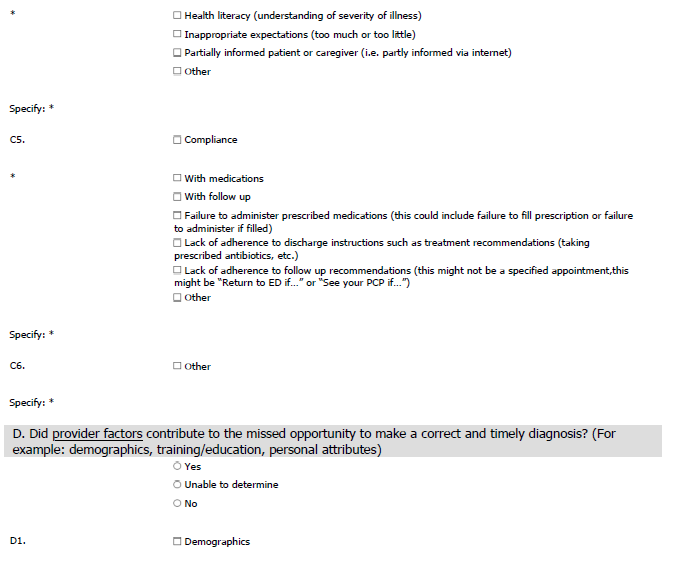


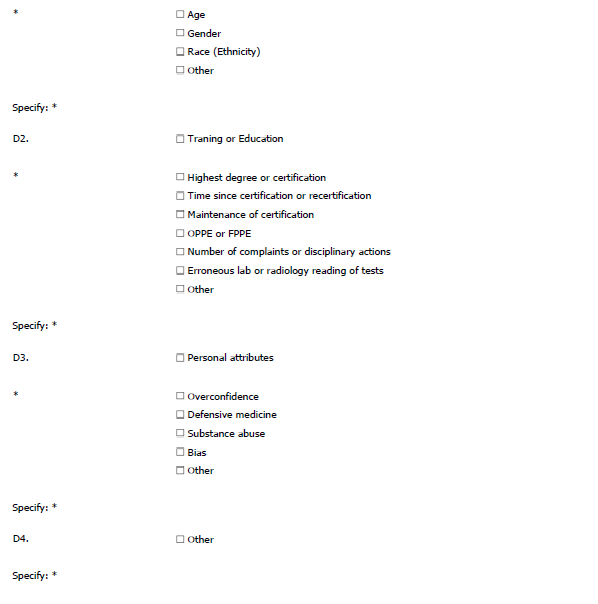


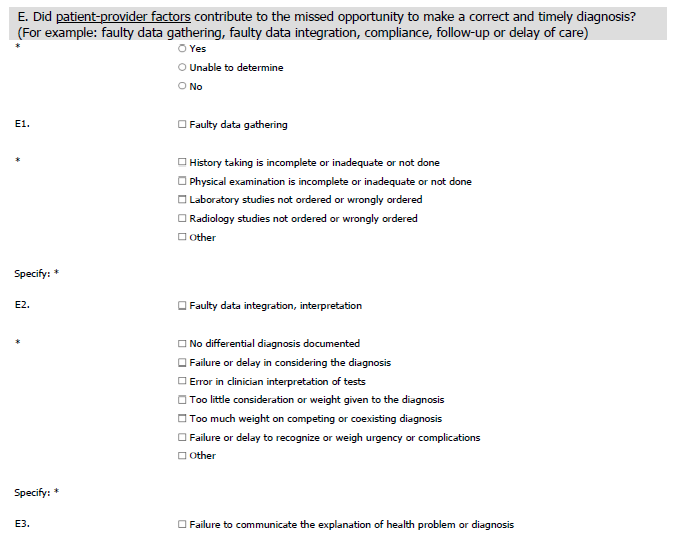


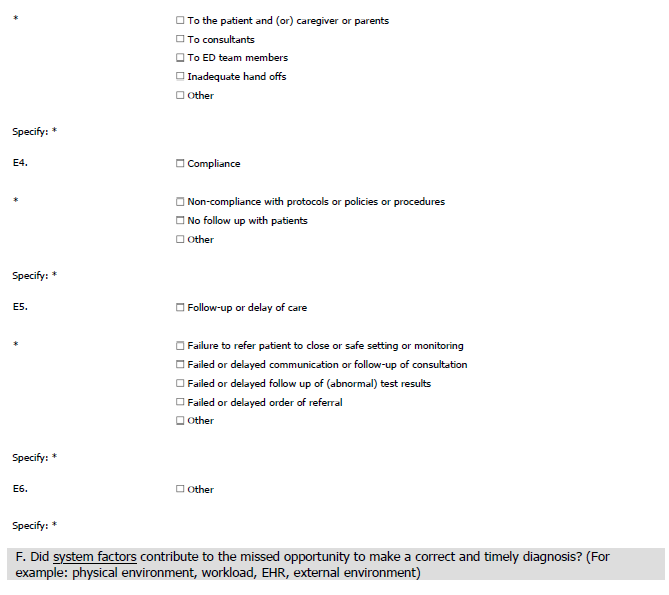


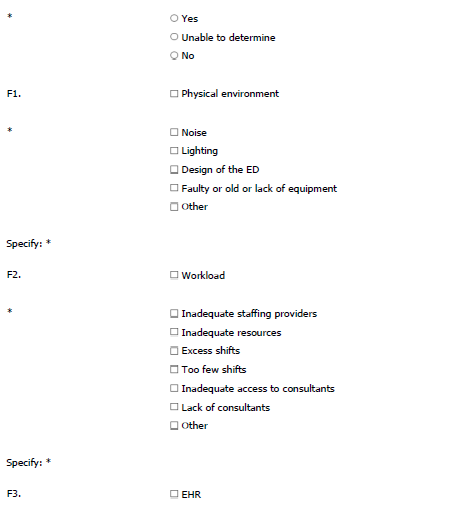


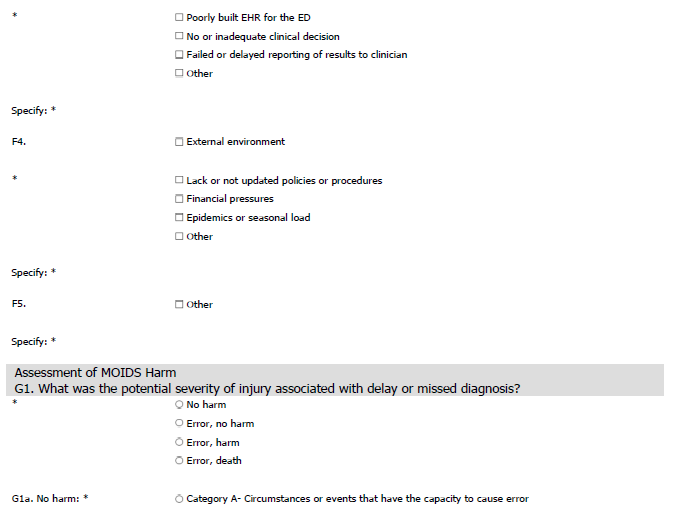

Supplement: Supplementary file 4 — Data S4. [file ACEM-32-226-s004.docx]
