# Supplementary material for: Epidemiology of diagnostic errors in pediatric emergency departments using electronic triggers
Source: Acad Emerg Med. 2025 Jan 15;32(3):226–45. doi: 10.1111/acem.15087 (PMC11921087; doi:10.1111/acem.15087)
Supplement: Supplementary file 5 — Data S5. [file ACEM-32-226-s005.docx]

**Supplementary material 4.** Reason for triggered chart exclusion as count and percentage overall and by trigger.

| **Reason excluded** | **Count** | **Percent of total** |
| --- | --- | --- |
| Appropriate ED care | 394 | 14.14% |
| Appropriate escalation | 335 | 12.02% |
| DOA or active CPR on arrival | 39 | 1.40% |
| Incorrect or limitation of programming | 98 | 3.52% |
| Progression of illness | 1612 | 57.86% |
| Psychiatric | 20 | 0.72% |
| Unrelated visits | 288 | 10.34% |
|  | **2786** | **100.00%** |

| **Reason excluded by trigger** | **Count** | **Percent by trigger** |
| --- | --- | --- |
| **1** | **1893** | **67.95%** |
| Appropriate ED care | 274 | 14.47% |
| Appropriate escalation | 66 | 3.49% |
| DOA or active CPR on arrival | 2 | 0.11% |
| Incorrect or limitation of programming | 47 | 2.48% |
| Progression of illness | 1196 | 63.18% |
| Psychiatric | 20 | 1.06% |
| Unrelated visits | 288 | 15.21% |
| **2** | **786** | **28.21%** |
| Appropriate ED care | 117 | 14.89% |
| Appropriate escalation | 267 | 33.97% |
| DOA or active CPR on arrival | 1 | 0.13% |
| Incorrect or limitation of programming | 31 | 3.94% |
| Progression of illness | 370 | 47.07% |
| **3** | **107** | **3.84%** |
| Appropriate ED care | 3 | 2.80% |
| Appropriate escalation | 2 | 1.87% |
| DOA or active CPR on arrival | 36 | 33.64% |
| Incorrect or limitation of programming | 20 | 18.69% |
| Progression of illness | 46 | 42.99% |
|  | **2786** | **100.00%** |
